# Taguatagua 3: A new late Pleistocene settlement in a highly suitable lacustrine habitat in central Chile (34°S)

**DOI:** 10.1371/journal.pone.0302465

**Published:** 2024-05-22

**Authors:** Rafael Labarca, Matías Frugone-Álvarez, Liz Vilches, José Francisco Blanco, Ángela Peñaloza, Carolina Godoy-Aguirre, Álvaro Lizama-Catalán, Cristóbal Oyarzo, Carlos Tornero, Erwin González-Guarda, Ayelen Delgado, Marcela Sepúlveda, Paula Soto-Huenchuman

**Affiliations:** 1 Facultad de Ciencias Sociales, Escuela de Antropología, Pontificia Universidad Católica de Chile, Santiago, Chile; 2 Facultad de Ciencias, Departamento de Química Ambiental, Universidad Católica de la Santísima Concepción, Concepción, Chile; 3 Formación Geológica SpA, Chimbarongo, Chile; 4 Sociedad Chilena de Arqueología, Santiago, Chile; 5 Facultad de Filosofía y Letras, Universidad Nacional de Cuyo, Mendoza, Argentina; 6 Independent Researcher, Santiago, Chile; 7 Department of Prehistory, Autonomous University of Barcelona (UAB), Bellaterra, Spain; 8 Institut Català de Paleoecologia Humana i Evolució Social (IPHES-CERCA), Tarragona, Spain; 9 Instituto de Ciencias de la Ingeniería, Universidad de O`Higgins, Rancagua, Chile; 10 Institute of Archaeology, University College London, London, United Kingdom; 11 Facultad de Ciencias Sociales, Departamento de Ciencias Sociales, Universidad de Tarapacá, Iquique, Chile; 12 Facultad de Ciencias, Laboratorio de Ontogenia y Filogenia, Red Paleontológica U-Chile, Universidad de Chile, Santiago, Chile; 13 Unidad de Patrimonio Paleontológico, Consejo de Monumentos Nacionales, Santiago, Chile; University of Michigan, UNITED STATES

## Abstract

We present the results of the excavations and analyses of the diverse and exceptional archaeological assemblage of Taguatagua 3, a new late Pleistocene site located in the ancient Tagua Tagua lake in Central Chile (34°S). The anthropogenic context is constrained in a coherently dated stratigraphic deposit which adds new information about the mobility, subsistence strategies, and settlement of the early hunter-gatherers of southern South America. The age model constructed, as well as radiocarbon dates obtained directly from a combustion structure, indicate that the human occupation occurred over a brief time span around 12,440–12,550 cal yr BP. Considering taphonomic, geoarchaeological, lithic, archaeobotanical, and zooarchaeological evidence, as well as the spatial distribution combined with ethnographic data, we interpret Taguatagua 3 as a logistic and temporary camp associated mainly with gomphothere hunting and butchering. Nevertheless, several other activities were carried out here as well, such as hide and/or bone preparation, small vertebrate and plant processing and consumption, and red ochre grinding. Botanical and eggshell remains suggest that the anthropic occupation occurred during the dry season. Considering the contemporaneous sites recorded in the basin, we conclude that the ancient Tagua Tagua lake was a key location along the region’s early hunter-gatherer mobility circuits. In this context, it acted as a recurrent hunting/scavenging place during the Late Pleistocene due to its abundant, diverse, and predictable resources.

## Introduction

In recent decades, we have witnessed an exponential growth of new data on the early peopling of the Americas, resulting from multidisciplinary approaches that typically combine archaeology, geology, paleoecology, paleontology, and genetics ([1–4] and references therein). These new contributions have challenged the old theoretical peopling models, presenting a more complex scenario in which, among other things, failed colonization processes, diverse subsistence strategies and the coexistence of different technologies, are expected to be found in a rapidly changing environment.

Probably, the most controversial issue that has sparked the hottest debates among scholars is the timing of the arrival of early hunter-gatherers to America. Some researchers state there is sufficient evidence to support a very early peopling of the continent (∼130,700 calibrated years before present, cal yr BP) [5,6]. However, most of the very early putative sites fall within the ∼33,000–19,000 cal yr BP time frame [2,7–10]. Although all these proposals have received considerable criticism from those in favor of a more recent peopling process (post Last Glacial Maximum, <18,000 cal yr BP) [11–16], this growing evidence cannot be completely dismissed, especially if we consider the alternative of failed colonization processes. Interestingly, genetic [17–19] and geological data [20–22] suggest that, independently of archaeological radiocarbon dates, humans would have crossed the Laurentide and Cordilleran ice sheets from Alaska sometime between 19,500 and 14,000 cal yr BP.

Leaving this debate aside, there is a general consensus that, around 14,000–13,000 cal yr BP, several areas of the Americas were populated ([4,14,23–26], but see [27]), and it is plausible to speculate about an exploration phase [28] with even earlier dates. [14] propose, based on the Summed Calibrated Probability Density of radiocarbon dates, that humans arrived in South America around 15,500 cal yr BP and that the population did not grow significantly until ∼12,500 cal yr BP, following the Antarctic glaciation (see also [14]). This increase in radiocarbon dating coincides, at least in the southern cone of South America, with the appearance and expansion of Fishtail Projectile Points (FPPs) [26].

Population growth during the Late Pleistocene-Holocene transition required the effective exploitation of the most productive areas, and the exploration and the subsequent colonization of second-order areas [29]. In this regard, the Ancient Tagua Tagua Lake (ATTL), located in central Chile (34° 30’ S1 Fig), offered archaeological data from two early sites: Taguatagua 1 (TT-1, [30,31]) and Taguatagua 2 (TT-2, [32]), with a robust chronological framework, extinct faunal assemblages, and human presence, inserted into this "later" phase of the continent’s initial settlement. TT-1 has recently been re-dated to ca. 12,600 cal yr BP [33] and was interpreted as a small residential camp where megamammal and small vertebrate processing and consumption occurred [33–35]. TT-2, with dates between ∼11,600–11,300 cal yr BP [32], is interpreted as an intensive gomphothere hunting/butchering site [35]. Both sites have high quality raw materials of exotic origin, supporting extensive round-trip mobility [35]. Considering this evidence, the ATTL offers an excellent opportunity to explore and discuss topics other than those relating solely to chronology, such as the intensity of occupation, settlement patterns, mobility, and subsistence, among others, on a local scale. To assess these, we have undertaken new surveys and excavations within the Tagua Tagua basin, furnishing evidence of precise human occupation processes during the late Pleistocene and resulting in the discovery of the Taguatagua 3 (TT-3) archaeological site in 2019.

Here, we offer a comprehensive synthesis of the various archaeological material evidence and natural proxies from TT-3, an archaeological site endowed with exceptional preservation conditions. Considering its open-air lacustrine characterization, we first center our discussion on the formation processes occurring at the site, evaluating the assemblages’ origin and mode of deposition from a taphonomic and geoarchaeological perspective. Secondly, we discuss the function and seasonal occupation of TT-3, and its relationship with other early sites identified within the basin. We also include information on other archaeological sites from the semi-arid region of Chile, to get a broader insight into the mobility and use of space by these early hunter-gatherers.

### Geographic location, geological setting and paleoenvironment

**Fig 1:**
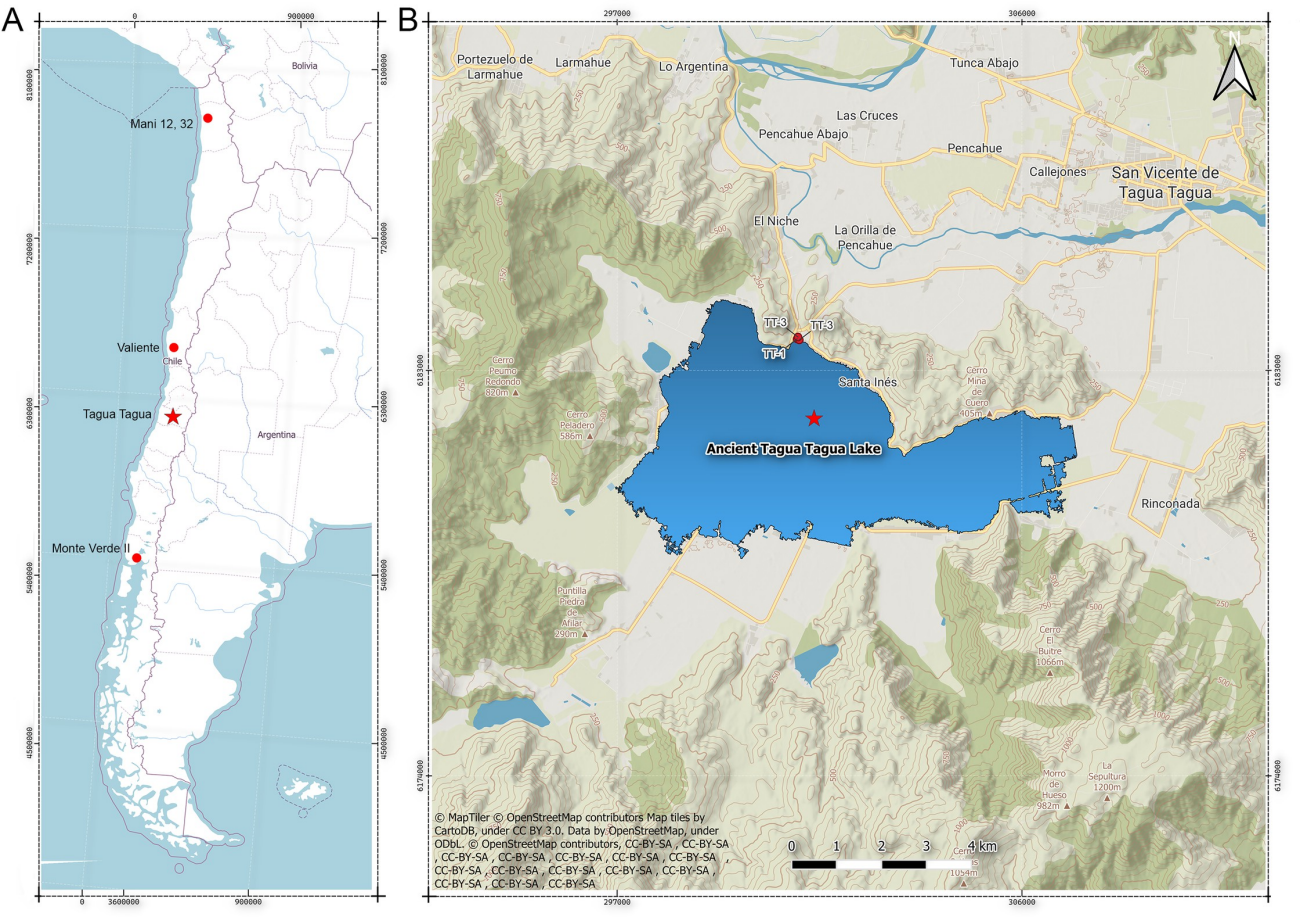
A. Location of the study area in a regional context and other early sites discussed in the text; B. Digital Elevation Model of the Tagua Tagua basin showing the locations of TT-1, TT-2, TT-3, the Valero-Garcés paleoclimatic column (red star) and the inferred maximum extension of the ATTL (see text).

**Fig 2:**
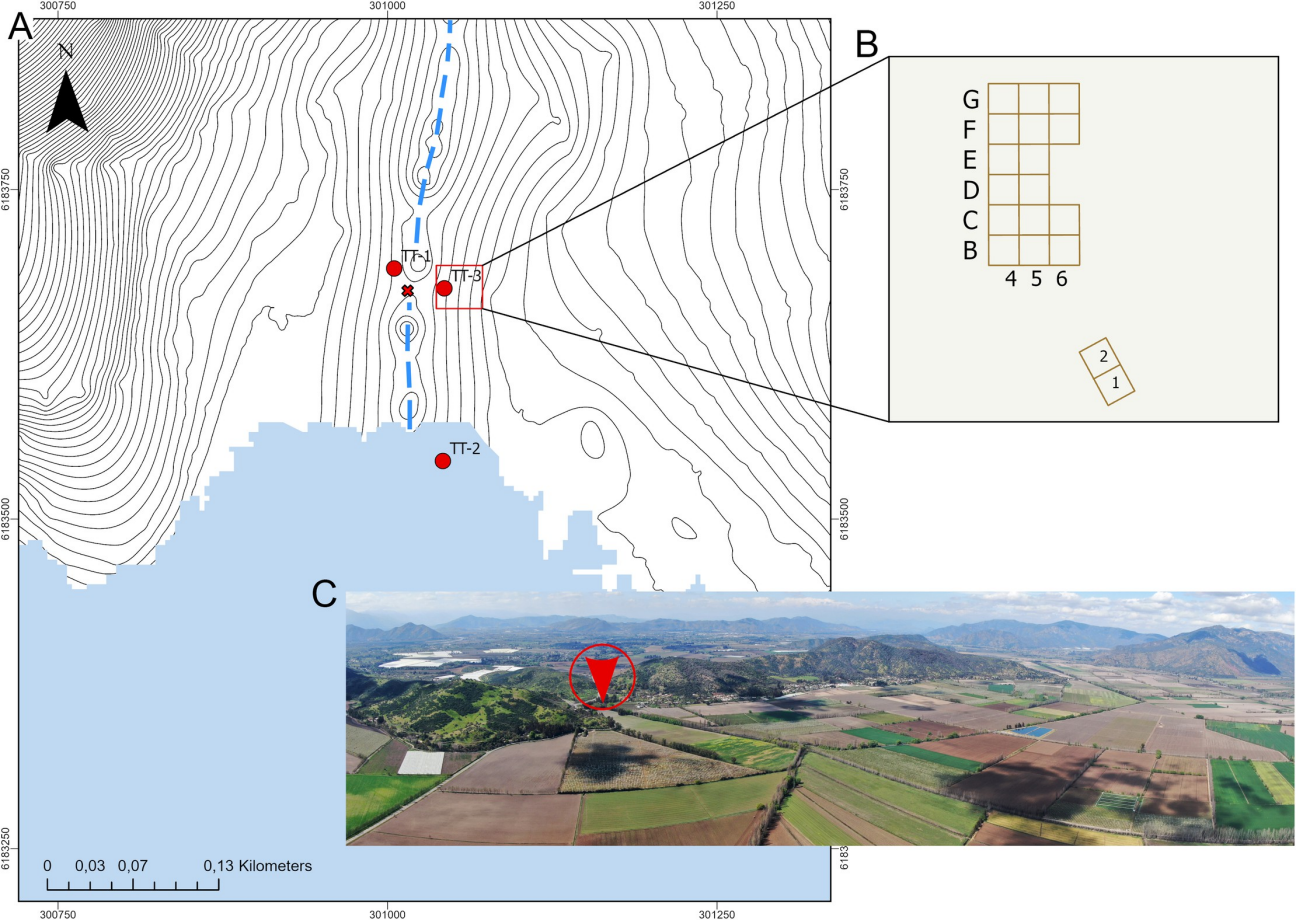
A. Detail of the location of early ATTL sites and Varela’s stratigraphic column (red star), considering a lake extension at 196 masl. The discontinuous light blue line indicates the drainage channel. B. Distribution of archaeological units excavated during 2019–2022 campaigns and location of the stratigraphic columns. C. Aerial view of the north side of the basin and the location of TT-3 (red arrow).

The ATTL is located about 5 km southeast of San Vicente de Tagua Tagua city, in a small closed intramontane basin of the Coastal Mountain range. It is an elliptical tectonic depression at about 196 meters above sea level (masl) delimited by a mountainous arch of ∼1000 masl with a small north-east facing opening (Fig 1). Until the mid-19^th^ century, the lake, which covered an area of ∼ 30 km^2^ and had a depth of about 5 m [36], was artificially drained for agricultural purposes [37], by means of an extensive channel cutting across the hills. Specifically, TT-3 is situated in the north area of the basin, in a “rinconada” or a small, subtriangular lake entrance, adjacent to the drainage channel, and very close to TT-1 and TT-2 (Fig 2).

**Fig 3:**
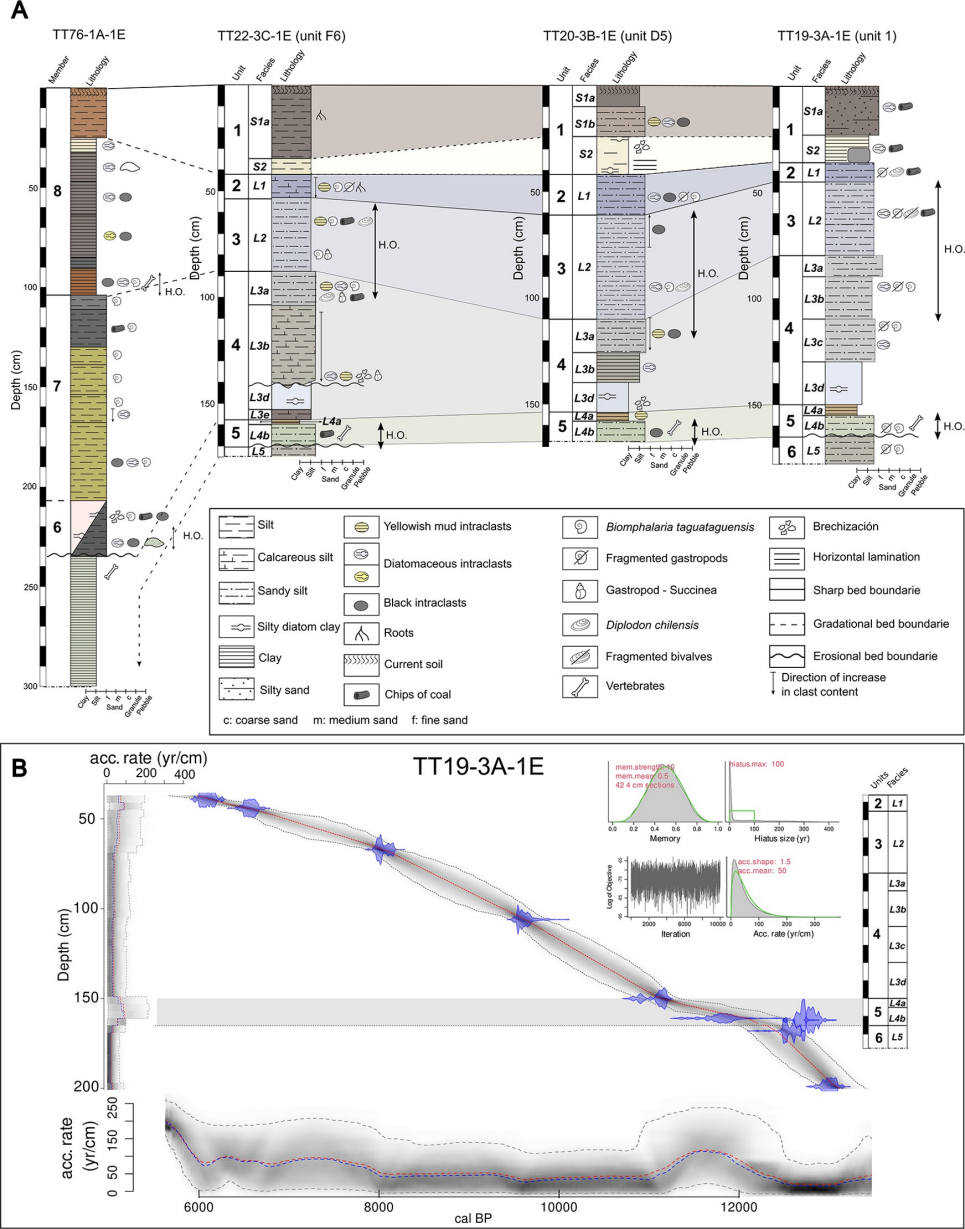
Correlation of the sedimentary chronosequences from north to south at the archaeological TT-3 site based on lithostratigraphic and sedimentological criteria, and the chronological model. A. Upper segment of the Laguna de Tagua Tagua Formation [43] and sedimentary sequences of TT-3: TT19-3A-1E (excavation unit 1; year 2019); TT20-3B-1E (excavation unit D5; 2020); TT22-3C-1E (excavation unit F6; 2022). B. Bayesian chronological model and accumulation rate (yr/cm) for the TT19-3A-1E sequence based on ten AMS ^14^C dates.

The Mesozoic basement rocks that underlie the basin form part of the La Lajuela Formation (Upper Jurassic–Lower Cretaceous) to the south, mainly of volcanic origin with some sedimentary intercalations, and the Alhué Plutonic Complex (Upper Cretaceous) to the north, of igneous, intrusive origin [38–41]. The basin is infilled by two semi-consolidated units, which from base to top comprise the Pudahuel Ignimbrite and Laguna de Tagua Tagua Formation (LTTF) [39,42]. Specifically, LTTF is divided into eight members, the first composed of sands and gravels of alluvial origin, while members 2 to 8 correspond to massive and/or laminated greenish to grayish gravelly clays, with some diatomaceous inclusions (Fig 3). While the top of the LTTF remains exposed as the current surface or recent soil [39,43], the age of its base is unknown, but member 3 was dated to around 40,200 cal yr BP [44]. Evidence of previously detected Late Pleistocene human occupation is situated at the erosive discordance between member 5 and 6 [43].

Central Chile has a semi-arid and Mediterranean climate influenced by the South Pacific Anticyclone (SPA) and the Southern Westerly Wind Belt (SWW), with strong oceanic control due to equatorward winds that force the upwelling of cold subsurface waters near the coast. Annual mean precipitation has a marked seasonality, largely concentrated in the austral winter (May–September), ranging from 100 mm to 1000 mm depending on altitude, latitude, and topography [41]. During the austral summer, the SPA prevails over the region, blocking the equatorward winds and causing dry conditions with some sporadic summer precipitation [45]. In the austral winter, precipitation is controlled by weakening of the SPA, which allows for frontal rainstorms to enter from the south. The Last Glacial Termination (∼18,000–11,700 cal yr BP) in the ATTL record is characterized by a progressive decrease in moisture, as indicated by increases in Chenopodiaceae, *Typha*, Gramineae, and Phorbiaceae, while arboreal taxa, such as *Nothofagus*, *Prumnopitys*, and Myrtaceae, decreased [46]. Drier conditions are also inferred from the sedimentological data and a δ^18^O peak suggesting a progressive lowering of the lake level [44]. However, these conditions are interrupted by a cold reversal event dated to between 13,500 and 11,500 cal yr BP [44]. The sedimentary record from Laguna del Maule (36°S, 2160 masl), located in the Andes of central Chile, shows a transition to wetter conditions between ca. 13,000 and 10,500 cal yr BP [47]. Early to mid-Holocene records indicate widespread warming in the mid-latitudes of the Southern Hemisphere between ∼11,500 and 8,000 cal yr BP, although inland moisture conditions are not entirely clear [44,47–52]. Based on the archaeological record to date, we assume that the environmental context, in which the first hunter-gatherers occupied this area around the ATTL, would have been cooler than today but sufficiently favorable to support a variety of species and resources for human exploitation.

## Results

### Chronostratigraphic framework

Sediments from the TT-3 archaeological site consist mainly of diatom-rich silt, as well as sandy and calcareous mud with abundant gastropod and bivalve remains. Based on their similarity in grain size, sedimentary texture, and biogeochemical data (see [53]), we have defined eight major facies grouped into six lithostratigraphic units (Figs 3 and S1). Five of these facies are of lacustrine origin (facies L; between units 6 and 2) and two are soil horizons (facies S; unit 1). Facies are arranged horizontally, and their boundaries are mainly gradational, suggesting a relatively constant sediment input on the sequence. Only the western portion of the excavation (units B4 to F4) were affected by landslides originating from the construction of the channel that drained the lake in the 19^th^ century. As a result, there is a limited abnormal westward and downward displacement of the sediments in this area, which did not alter the position of the cultural material within the facies (see S2 File for a detailed description of this feature).

All the lithostratigraphic units defined in TT-3 columns correlate well with members 5 and 8 described in the original LTTF sequence ([43], Fig 3A, labeled TT76-1A-1E), although the units defined in Varela’s original sedimentary description are thicker. Based on our Bayesian age-depth model (Fig 3B) constructed from ten AMS-radiocarbon dates (Table 1), the TT19-3A-1E sequence spans ∼13,000 cal yr BP (the mean 95% confidence ranges are 620 yr, min. 300 yr at 39 cm, max. 935 yr at 157 cm). Between Units 5 and 6, the sequence is interrupted by a hiatus (at 165 cm) with a length of 100 years estimated from the age-depth model.

**Table 1:** Radiocarbon dates from the TT-3 site.

Provenience	Depth cm	Facies	Dated material	Lab. Code	Date	2 σ cal BP	Comments
Exc. Unit 1/East profile*	039–040	L1	Charcoal	D-AMS 039542	5345±28	5992–6199	Near the top
Exc. unit 1/East profile*	044–045	L1	Charcoal	D-AMS 039543	5806±33	6481–6667	Near the top
Exc. unit 1/East profile*	067–068	L2	Charcoal	D-AMS 039544	7259±32	7958–8169	Near the top
Exc. unit 1/East profile*	106–107	L3b	Charcoal	D-AMS 039545	8657±45	9524–9700	Near the top
Exc. unit 1/East profile*	150–151	L3d	Bulk sediment	D-AMS 039546	9736±36	10870–11222	Near the top
Exc. unit 1/East profile*	161–162	L4b	Bulk sediment	D-AMS 039547	10211±63	11619–12061	Near the top
Exc. unit 1/East profile*	162–163	L4b	Charcoal	D-AMS 039548	10936±41	12747–12902	Near the top
Exc. unit 1/East profile*	162–163	L4b	Bulk sediment	D-AMS 039549	10747±49	12627–12746	Near the top
Exc. unit 1/East profile*	168–169	L4b	Charcoal	D-AMS 039550	10590±39	12490–12620	Middle
Exc. unit 1/East profile*	199–200	L5	Bulk Sediment	D-AMS 039551	11117±51	12897–13104	
Exc. unit 1	040–50	L1	Charcoal	D-AMS 038097	5250±31	5904–6017	
Exc. unit G5	79	L3b	Charcoal	D-AMS 051771	7668±33	8370–8520	Near the top
Exc. unit G5	102	L3b	Charcoal	D-AMS 051769	8096±42	8760–9036	Near the base
Exc. unit C5	175	L4b	Charcoal	D-AMS 043769	10572±39	12474–12635	Combustion feature (middle)
Exc. unit C5	177	L4b	Charcoal	D-AMS 043771	10503±36	12260–12613	Combustion feature (base)
Exc. unit 1	170–180	L4b	Charcoal	D-AMS 038100	10536±39	12456–12624	

*: Used for age model.

Unit 6 (facies L5), located at the base of sequences TT22-3C-1E and TT19-3A-1E, corresponds to well-consolidated, grayish-green, sandy silts with poorly selected clasts ranging from granules to cobbles (Fig 3A). This unit spans between ∼13,100 and 12,700 cal yr BP with a median sediment accumulation rate of ∼60 yr/cm^−1^. Units 5 (facies L4b and L4a), which spans between ∼12,700 and 11,160 cal yr BP with a median accumulation rate of ∼100 yr/cm^−1^, corresponds to two intervals of homogeneous diatomaceous sediments rich in inorganic carbon. Specifically, facies L4b was defined as a greenish to gray, semi consolidated, sandy silt with complete and fragmented gastropods at the base and abundant charcoal spicules (10 to 17 cm thickness). This facies contains the earliest human occupation at its base and spans between ∼12,700 and 11,500 cal yr BP. Facies L4a consists of dark brown, fine-grained, massive, inorganic carbon and diatom-rich silt with a maximum thickness of 5 cm. The term "Paleo-soil" was originally employed by [43] to name this facies, but current biogeochemical data indicates a lacustrine origin [53]. Facies L4a span between ∼11,500 and 11,160 cal yr BP. Unit 4 (facies L3a, b, c, d, and e) consists of massive sandy silt of gray-green to grayish lacustrine diatom ooze facies with abundant intraclasts of yellowish mud and diatom fragments. These facies have variable grain sizes with diverse assemblages of diatoms, sponge spicules, and phytoliths. Intraclasts are dominant from the base of this unit, while gastropod remains are more abundant at the top. L3 has a thickness of between 0.7 and 0.42 m and spans between 11,160 and 8,500 cal yr BP with a median rate of ∼60 yr/cm^−1^. This facies is archaeologically fertile at its top. Unit 3 is formed only by facies L2 with ages between ∼8,500 and 6,600 cal yr BP and median accumulation rate of ∼100 yr/cm^−1^. This unit is composed of gray sandy and calcareous silts, with a thickness of 0.3 and 0.48 m. It includes the gastropods *Biomphalaria taguataguensis* and *Succinea* sp. as well as the bivalve *Diplodon* sp. Diatomaceous and yellowish mud intraclasts and charcoal spicules were also found. This facies is archaeologically fertile. It was affected by tree roots and burrows particularly in the upper half of the western area. Unit 2 is composed of the lacustrine facies L1 and spans between 6,600 and 6,000 cal yr BP with a median rate of ∼150 yr/cm^−1^. This facies is a massive dark gray, calcareous and sandy silt with intraclasts of yellowish mud that increase toward the base. *B*. *taguataguensis* shells, both complete and fragmented, are present. The facies is bioturbed by roots and lagomorph burrows, spatially to the west of the excavated area. The thickness of the L1 facies varies between 0.07 and 0.18 m. Finally, Unit 1 (Facies S1 and S2, between 0 and 40 cm) is composed of a massive dark to brownish silt, with diatomaceous and black intraclasts. It contains subactual cultural material and shows clear evidence of agricultural disturbance but was not included in the age model. Facies S2 consist of grayish to white, feasible, diatomaceous clay with charcoal spicules and diatomaceous ooze banks, with a variable thickness of 0.05 and 0.15 m. The top of S1 corresponds to the actual surface and varies in thickness between 0.23 and 0.35 m.

The results obtained enable a stratigraphic correlation between TT-1 and TT-3 Pleistocene anthropogenic occupations. The published stratigraphic column from TT-2 [32] could not be satisfactorily correlated.

### Vertical distribution

**Fig 4:**
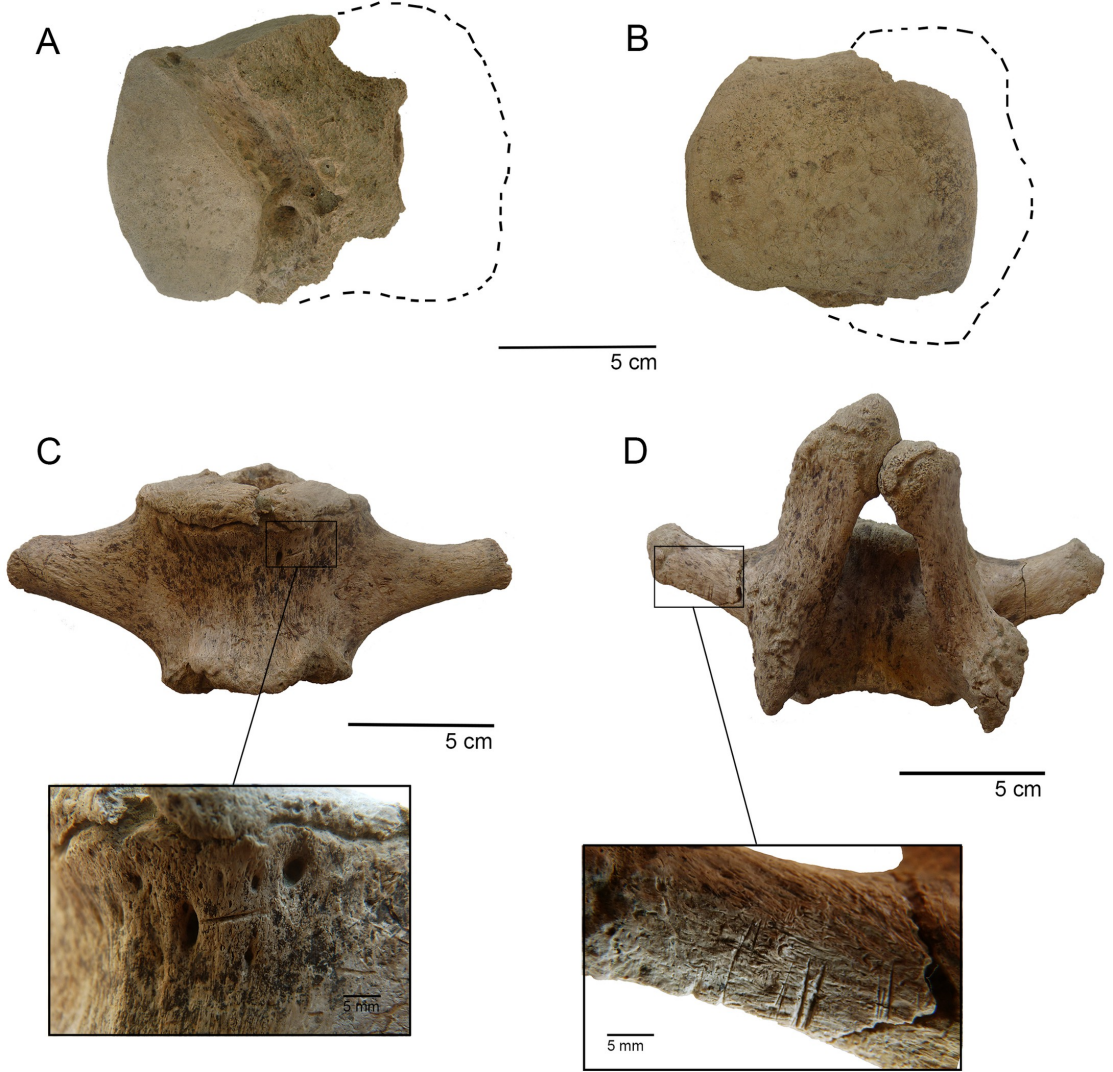
Relative frequencies of faunal remains and taphonomic modifications. A. Small vertebrate distribution on facies L4b; B. Taphonomic modifications on the lower level of facies L4b. C. Manganese intensity on the lower level of facies L4b; D. Fire intensity on the lower level of facies L4b. Manganese intensity was recorded as follows: Stage 0: unmodified; Stage 1: Light (<25% of the surface covered); Stage 2: Moderate (<50% of the surface covered); Stage 4: Severe <75% of the surface covered); Stage 5: Extreme (100% of the surface covered). Chromatic stages of fire action were recorded as follows: Stage 0: unmodified; Stage 1: mostly brown; Stage 2: mostly black; Stage 3: mostly gray/white.

There is a marked difference in the amount and diversity of archaeological material among the upper and lower level of facies L4b. Regarding the faunal remains (NSP = 20,353), only 28.7% of the specimens come from the upper level. There are significant differences (x^2^ = 760.8; df = 5; p<0.001) in the frequencies of the diverse taxonomic groups identified. For example, nearly all megafauna remains come from the lower level. Although some bigger megafauna fossils began to appear in the upper level, they always rested at the base of facies L4b. The lower level also contains, comparatively, the highest frequencies of birds and rodents; and, inversely, fish and amphibians are more common in the upper level (Table 2 and Fig 4A). Small vertebrates with cutmarks were detected only in the lower level, while burned bones appeared in both levels, being more common in the lower one (see below). Lithic material is also more common in the lower level. In fact, only three of the fifteen specimens (TT3-G6-N17-01; TT3-F6-N17-02 and TT3-G5-N17-02) were found in the upper level, but near its base. Also, in the lower level, an *in-situ* combustion feature was recovered, with its base at precisely the end of the facies L4b (see below).

**Table 2:** Summary of faunal remains from facies L4b at TT-3.

		Upper level		Lower level		
Taxa	NISP	MNE	MNI	NISP	MNE	MNI
Gomphotheriidae undet. (Proboscidea)	265	1	1	8374	20+	1
*Equus* sp. (Equidae)	0	0	0	1	1	1
*Antifer ultra* (Cervidae)	0	0	0	1	1	1
Artiodactyla undet.	0	0	0	1	1	1
Rodentia undet.	103	*	*	816	*	*
Aves undet.	310	*	*	910	*	*
Amphibia undet.	1,234	*	*	775	*	*
Reptilia undet.	0	0	0	1	1	1
Actinopterygii undet.	2,268	*	*	954	*	*
Small vertebrates undet.	1,669	*	*	2,671	*	*
Total	5,849	*	*	14,504	*	*

* Not calculated for the entire sample. + Vertebral discs associated with their respective vertebrae were counted as a single element.

We propose, therefore, that the early cultural activity at the site occurred during the beginning of the deposition of facies L4b (roughly the lower level). The latter justifies an analytical segregation between the upper and lower levels, especially regarding a faunal analysis of small vertebrates, considering their representation in the total sample.

### Faunal remains: Taxonomy, frequencies of skeletal parts and taphonomy

Fossil bones are the most common material recovered in facies L4b. We have centered our description and analysis on the lower level, but the upper level is referred to mainly for comparative purposes. In the former, megafaunal remains comprise nearly 60% of the total sample. If this subset is taxonomically segmented, the Family Gomphotheriidae is the most popular taxa, but is represented by few elements (S1 Table). Remains are not diagnostic of a generic or specific level, but the morphology of the molars in the sample (i.e., bunodont, with a well-developed central line, and thrilophodont in the case of intermediate teeth) are diagnostic of the Family (i.e., [54–56]). The genus *Equus* is represented only by a first phalanx. The fossil is elongated, with its body latero-medially compressed. In the posterior view, the *trigonium phalangis* is V-shaped, exceeding half the length of the phalanx. This trait is diagnostic of the *Equus* genus, since, in *Hippidion*, this tuberosity is short and segmented into two [57,58] (S2 Fig). Finally, the apex of a tine’s antler assigned to *Antifer ultra* and the dorsal portion of a rib of an undetermined Artiodactyla were also identified.

Small vertebrates are represented by the Order Rodentia, Reptilia and Amphibia, and the Class Aves and Actinopterygii. Among the rodents, it is possible to determine preliminarily almost exclusively the presence of members of the family Octodontidae, while in Aves members of the Order Gruiformes, Passeriformes, Podicipediformes, Anseriformes, Charadriiformes, and Columbiformes have been tentatively identified. Nevertheless, anatids (Anseriformes) seem to be the most predominant group. Regarding amphibians, diagnostic cranial remains (i.e., ornamented frontoparietals) of *Calyptocephallela* sp. have been recovered. Bones from smaller taxa (i.e., Bufonidae) were recovered in much lower quantities.

Determined proboscidean fossils at the lower level correspond mainly to axial elements of a single individual. A collapsed skull is represented by more than 6,500 thick pneumatized bone fragments of different sizes. Some recognizable specimens include maxillary fragments and molar alveoli. Associated with the latter, five molar fragments were recorded which may correspond to four elements. The presence of a completely worn DP4, and possibly a M1 without wear (at least in its second and third lophids) suggest an immature individual (around 7 to 12 years old) or a very young subadult (12.5–15 years old) at the time of death, according to [56]. Tusks are absent. The vertebral column is represented by a cervical vertebra, a lumbar vertebra, four sacral vertebrae, and four caudal vertebrae. Some vertebral discs were also recovered, always detached from their bodies. Also, several portions of unfused coxal portions (i.e., coxal tuberosity, ischiatic tuberosity) and ridges of the iliac wing were identified. From the appendicular skeleton, a right astragalus, a right carpal 3, and a proximal sesamoid were recovered. Fossil remains of the upper level are scarce and correspond mainly to small cranial remains coming from excavation unit C5 (S1 Table).

Small vertebrate remains in the lower level of facies L4b comprise 42.24% of the entire faunal sample. Fish are the most abundant taxa (15.57% of small vertebrates), but these values are lower compared to the upper level (40.61% of small vertebrates). Birds and rodents are also abundant (14.85% and 13.31% of small vertebrates respectively), and their proportions are higher compared with the upper level (5.5% and 1.8% of the small vertebrates respectively). Amphibians are the least represented taxa in the lower level (12.64% of the small vertebrates) but in the upper one, they are the second most recorded taxa (22.09% of small vertebrates) (Table 2 and Fig 4A).

Regarding rodents, a total of 13 individuals were estimated from the lower level of excavation units C5, E4 and E5. Both the %MAU and %RA indicate that the skull and mandible are well represented (>60% in both measures), as well as long bones and the pelvic girdle (>53% in both measures). Comparatively, vertebrae and ribs are less predominant (S2 Table). Following Andrew’s indexes (1990), the rodent samples present a higher number of postcranial than cranial elements (1.27), nearly the same proportion of cranial and proximal limbs (109) and more proximal than distal limbs (0.71). With respect to birds, there was an MNI of six individuals considering all the orders identified in the subsample. %MAU and %RA are consistent and showed that cranial and mandibular bones, excluding the quadrate bone, have a low representation. In contrast, vertebrae, and particularly thoracic ones, are well represented (>57% in both measures). With respect to the pectoral girdle, scapula and sterna are better represented than furcula and coracoids (S3 Table). Finally, there is a higher prevalence of leg bones than wing bones (>%57 in femur, tibiotarsus, and carpometacarpus). The latter is confirmed by the wing-to-leg ratio (0.4). Additionally, there is a similar proportion of proximal and distal limb bones (0.5), and more limb bones in relation to core bones (0.32). Amphibians are represented by at least nine individuals in the subsample: three subadults, three adults and three indeterminate. Although the samples are small, a total of 17 individuals were calculated from the upper level, of which 12 are juveniles and only one is an adult. In the lower level, the sample is dominated by cranial bones, especially frontoparietals and exooccipitals. Mandibles and pelvises are also well represented (%MAU >43). Limb bones are scarce (S4 Table). If [59] indexes are considered, the lower level is characterized by a marked predominance of cranial bones (0.28), more anterior than posterior limb bones (1.16) and more distal than proximal ones (1.16). In fish, three individuals were identified. Frequency of the skeletal part is dominated by vertebrae, especially thoracic, and neurocranium bones (%MAU > 50). Facial bones are comparatively less well represented (S5 Table).

The MNE/NISP ratio (0.002) for the proboscidean sample taken from the lower level suggests a very fragmented assemblage, but the presence of the highly fractured skull and other isolated small fragments ambiguate this value (S1 Table). The remaining fossils are complete or nearly complete. In vertebrae, only their weakest portions are either missing (i.e., a portion of the transverse process) or were recovered completely but broken *in situ* (i.e., body is separated from the arch). The same is true of the vertebral discs and pelvic fragments, portions of which—in all cases—were recovered in several associated specimens. In these cases, the fractures’ morphology and outline indicate that fragmentation occurred with the bone in dry state. Other specimens, such as gomphothere’s feet and manus bones, and even the horse’s first phalanx, were recovered complete. Skull fragmentation could be associated with several factors and agents including sediment pressure, human manipulation (either due to deliberate breakage or fire exposure), and other natural geological processes.

**Fig 5:**
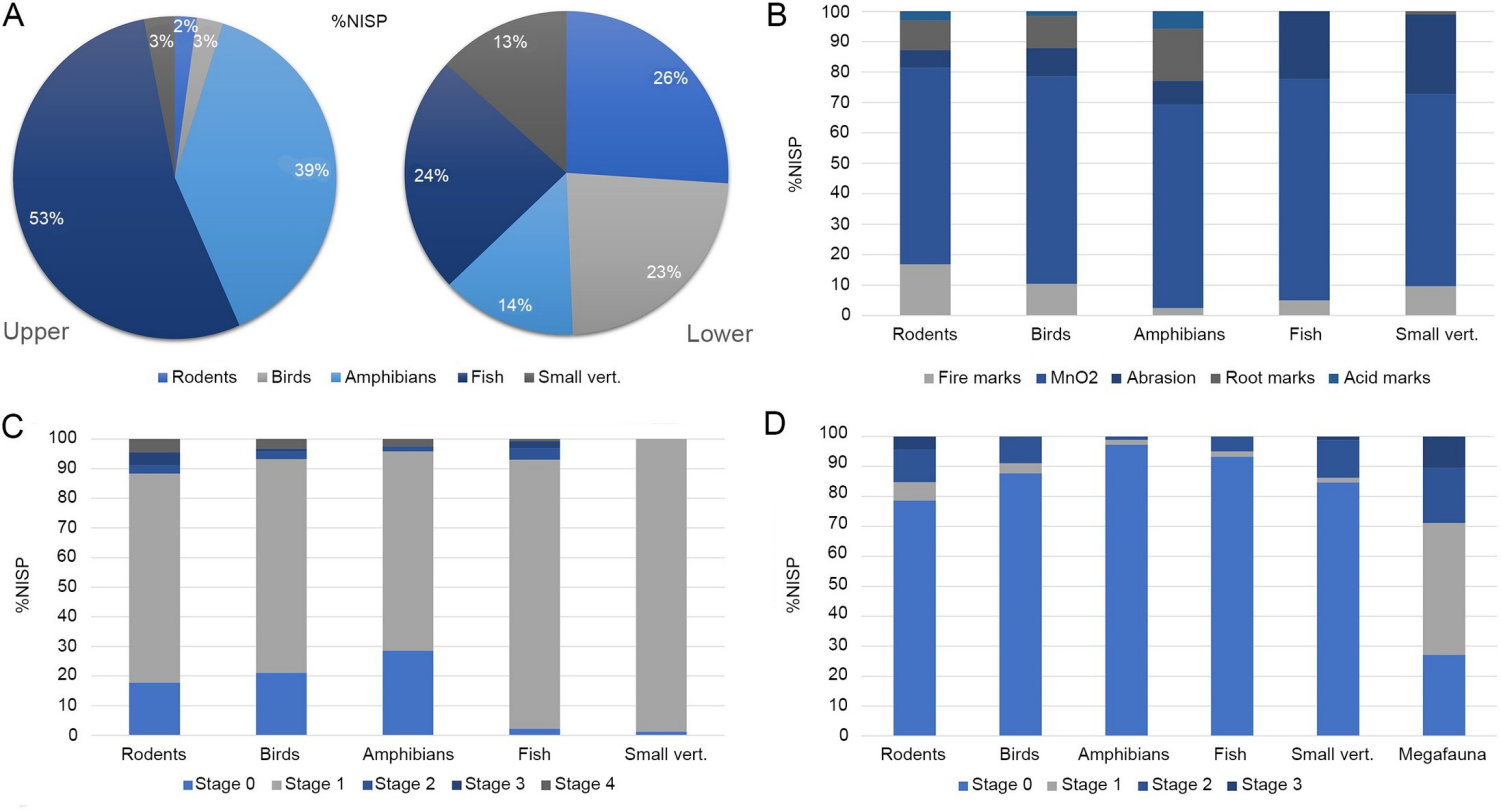
Taphonomic modification on gomphothere bones. A-B. Astragalus (TT3-E5-N18-06) with tooth marks in ventral and dorsal view (discontinued line indicates the original extension of the specimen); C. caudal vertebra (TT3-E4-N18/19-02) with a single cutmark; D. caudal vertebra (TT3-E5/F5-N18-53) with several parallel cutmarks.

Megafauna bones are not weathered, and only one fossil exhibits carnivore tooth marks. Extensive furrowing and several big scorings (i.e., 7–8 mm wide in its minor axis, [60] were observed in lateral and dorsal areas of the calcaneus (TT3-E5-N18-06). In fact, the tuberosity is completely absent and nearly all the lateral (ectal) facet was consumed. The tibial facet is incomplete as well. Various pittings were recorded in the latter sector (Fig 5A and 5B). The intensity and dimensions of the modifications rule out the actions of a small carnivore (such as a fox); instead, it is compatible with a large feline [60–63]. No trampling marks, abrasion, rodent marks, or gastric corrosion were detected on the megafauna sample.

Taphonomic modifications following the bones’ burial are comparatively more common. Root etching, for example, was detected in 57.9% of the megafauna MNE. However, root action is spatially constricted on the fossils and only affects their surface, without creating deep grooves. Manganese coating is observed in 100% of the megafauna MNE but is characterized mainly by isolated blackish spots on the fossils, in some cases forming very specific coated areas, without covering the specimens significantly (i.e., <40% of the fossil’s surface). The only exception is the horse phalanx, which is heavily coated (S2 Fig).

Two types of anthropogenic modifications were detected in the megafaunal subsample: cutmarks and fire marks. The incidence of fire in the lower level of facies L4b subsample is 78.2% (NISP = 3098) and was detected almost exclusively on small cranial or unidentified gomphothere fragments. It is interesting to note that some cranial fragments exhibit fire marks on their inner surface, suggesting that fire acted before fragmentation. Cervical vertebrae and a M1 exhibit localized fire action, which is coherent with its spatial location near to the anthropogenic hearth (see below). Nevertheless, unidentified burned bones were recovered not only in C5, but also in E4 and E5. Chromatically, Stage 1 (brown) was the most abundant (NISP = 1,870; %NISP total = 44%). On the other hand, cutmarks were registered exclusively in three proboscidean elements, all of them caudal vertebrae. The first element (TT3-E4-N18/19-02) possesses a single, short cutmark (75 mm long) located on the caudo-ventral portion of the body (Fig 5C); in a second caudal vertebra (TT-3-F4-N18/19-02), a single long cutmark (216 mm long) was detected at the base of the vertebral arch, being cranio-caudally oriented. Finally, the specimen TT3-E5/F5-N18-53 possesses 11 parallel cutmarks in the dorsal area of the right transverse process forming three groups of different longitudes and depths (Fig 5D).

In summary, the low proportion of natural modifications that occur in subaerial conditions (i.e., weathering, tooth marks, and rodent marks), suggests that the proboscidean bone assemblage was not extensively exposed prior to its burial. Nevertheless, an anthropogenic manipulation of the carcasses was confidently detected, as well as a minimal carnivore intervention. Fractures could be related mainly to geological processes that occurred *in situ* after the megafaunal sample was buried, but human agency on the fragmentation of the gomphothere skull cannot be ruled out. Other marks observed, such as root etching and manganese, are compatible with a wet environment deposition [64] which occurred after the burial of the fossil sample.

In small vertebrates (archaeological units C5, E4 and E5), the most frequent natural modification in the lower level is MnO_2_ (%NISP = 87.5) (Fig 4B and S6 Table). Fish are the most affected taxa (%NISP = 97.8). In contrast, birds are the least affected (%NISP = 79.7%). The percentage of manganese coating on these fossils is also variable, but the Stage 1 (<15% of the surface covered) showed the highest value (%NISP = 80) considering all small taxa but also on each taxon separately (S7 Table). As in the megafauna sample, the low proportion of surface covered with manganese did not impact the observation of other taphonomic modifications (Fig 4C). Natural abrasion was the second most recorded modification, as reflected in polished surfaces and/or rounded fracture edges. Nearly a third of the fish sample exhibits this feature (%NISP = 30%), while only 7.4% of the rodent assemblage presented some kind of abrasion (Fig 4B). It’s difficult to link this modification with any specific agent, nevertheless, considering the low proportion of gastric corrosion (i.e., pitting) and the absence of a sandy substrate, we hypothesize water abrasion acting *in situ* with the bones already buried. Root etching (%NISP = 9.2) presents a very particular pattern of distribution, since it was recorded exclusively in rodents, amphibians, and birds. Root marks do not completely cover the bone, being found only at specific points of the specimens. Amphibians were the more affected taxa (%NISP = 20.8) and rodents the least (%NISP = 12.4) (Fig 4B and S6 Table).

Gastric corrosion (i.e., pitting) was scarce, with only 2.3% of the assemblage altered in the lower level (Fig 4B and S6 Table). Again, the fish assemblage did not present such modifications. Amphibians were the most corroded group (%NISP = 7; MNI = 3, all juveniles), and birds were comparatively less affected (birds: %NISP = 1.8; MNI = 2; rodents: %NISP = 3.8; MNI = 2). Regarding intensity, the light category was the most predominant (%NISP = 64.4), but moderate and severe corrosion were also recorded (%NISP = 24.4 and 11.1, respectively). Even considering the small size of the sample, some differences were observed among taxa. The severe category was recorded only in amphibians (NISP = 5; %NISP = 27.7), whilst the moderate category exhibits the highest value in this group (NISP = 6; %NISP = 33.3%). On the other hand, only light and moderate categories were recorded for both rodents and birds; the latter (NISP = 3; %NISP = 15.8 and NISP = 2; %NISP = 25, respectively) is less represented than the former (S8 Table).

**Fig 6:**
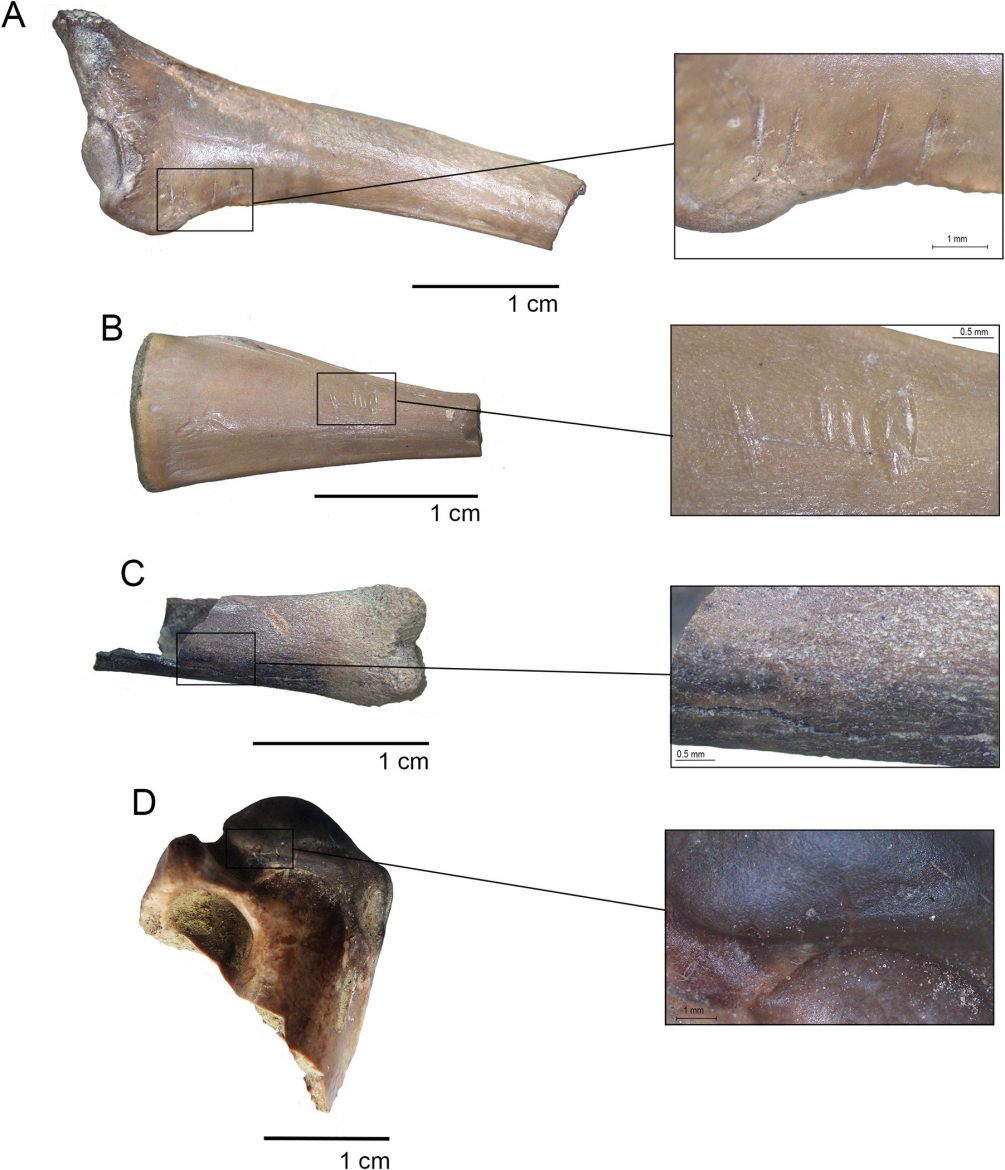
Anthropic modifications on small vertebrate specimens. A. cutmarks on a scapula of Anatidae; B. cutmarks on the fibular tarsal of Anura; D. fire marks on the distal femur of Caviomorpha; D. fire marks on a proximal humerus of Anatidae.

Alterations associated with fire exposure were observed in 12.5% of the small vertebrate subsample in the lower level and were detected across all the taxonomic groups (Fig 4B). Comparatively, in the upper level, 7.8% of the entire subsample presents this modification. Rodent is the most burned group (%NISP = 21.4; MNI = 3), followed by birds (%NISP = 12.3; MNI = 3), fish (%NISP = 6.7; MNI = 1) and amphibians (%NISP = 3.1; MNI = 1). In all taxa but Amphibia, Stage 3 (blackish coloration) is the most represented category (ranging from 50.5% of the burned NISP in rodents to 70.9% in birds and fish). Only in rodents, Stage 2 (brownish coloration) and Stage 4/5 (grayish/whitish coloration) were proportionally important (%NISP of burned bones = 22.4 and 20.6 respectively) (Figs 4D, 6C and 6D). The distribution of fire marks in rodent skeletons does not show a clear pattern. Nearly all the identified elements show some degree of fire modification, nevertheless, the most burned bones (burned MNE/total MNE) are lumbar and caudal vertebrae, and to a lesser extent, pelvises and tibiae (S2 Table). Interestingly, bones that are very well represented such as crania, mandible, ulnae, femora, calcanei, and humeri do not show high frequencies of burning. In this regard, the correlation between burned %MAU and %MNE is not significant (rs = 0.34, p = 0.075). Due to the fragmented condition of the sample, it was impossible to evaluate whether the bones were originally completely burned or if only a portion of them were exposed to fire (i.e., articulated and/or with soft tissue). Spatially, it is remarkable that the proportion of burned bones is similar among the three excavation units analyzed (C5 = 20.7%; E4 = 20.9%; E5 = 22%). In birds, most burned bones are coracoids and femora, but the samples are small. Vertebrae, which are well represented in the sample, exhibit a low proportion of burning. In fish, most of the burnt bones (NISP = 20) correspond to unidentified remains.

Two specimens present cutmarks, both recovered in the lower level from excavation unit C5. A bird scapulae presented human traces in their glenoid cavity, while a single amphibian tarsal-fibular showed anthropogenic modifications at the distal portion of the diaphysis, on the dorsal side (Fig 6A and 6B).

In summary, taphonomic modification observed in the small vertebrate subsample suggests a very complex depositional scenario. Several processes altered them, some in very specific points of its taphonomic history (i.e., tooth marks, cutmarks), but others probably affecting the sample during prolonged periods of time and/or in a cyclic way (water abrasion, root etching). We interpret that the small vertebrate assemblage was deposited by three main agents (not mutually exclusive): human, non-human predators, and natural deaths.

### Eggshells

Two of the three archaeological egg specimens analyzed, both from the lower level of facies L4b, were identified as Anatidae undet. due to the presence of marked pores, an irregular arrangement of mammillae, and angular membrane facet. The thickness of the sample (X = 0.4 mm) is greater than the reference specimens (X = 0.29 mm). There are some morphological and distributional differences between both samples (i.e., number of principal pores, arrangement, and distance between mammillae), which suggest the presence of two taxa (S3 Fig). Some minor taphonomic modifications were observed (i.e., some eroded areas). No fire marks were detected.

### Lithic material

The lithic assemblage is characterized by three main groups of raw materials: local rocks identified as coarse grained porphyritic-textured andesites and basalts (n = 8; 53.3%); possibly exotic rocks of sedimentary origin, labeled with the generic term “siliceous” (n = 6; 40%; and an exotic black obsidian with vitreous texture (n = 1; 6.6%). The source of this latter material has been evaluated using XRF analysis, with values that do not match any published sources [65–67]. However, judging by the similitude obtained for the Sr/Rb ratio (S4 Fig), it could be tentatively associated with the Maule group, which has several primary sources, relatively unknown to date [65,68,69]. This source group is found about 100 km south of TT-3, at Laguna del Maule [70].

**Fig 7:**
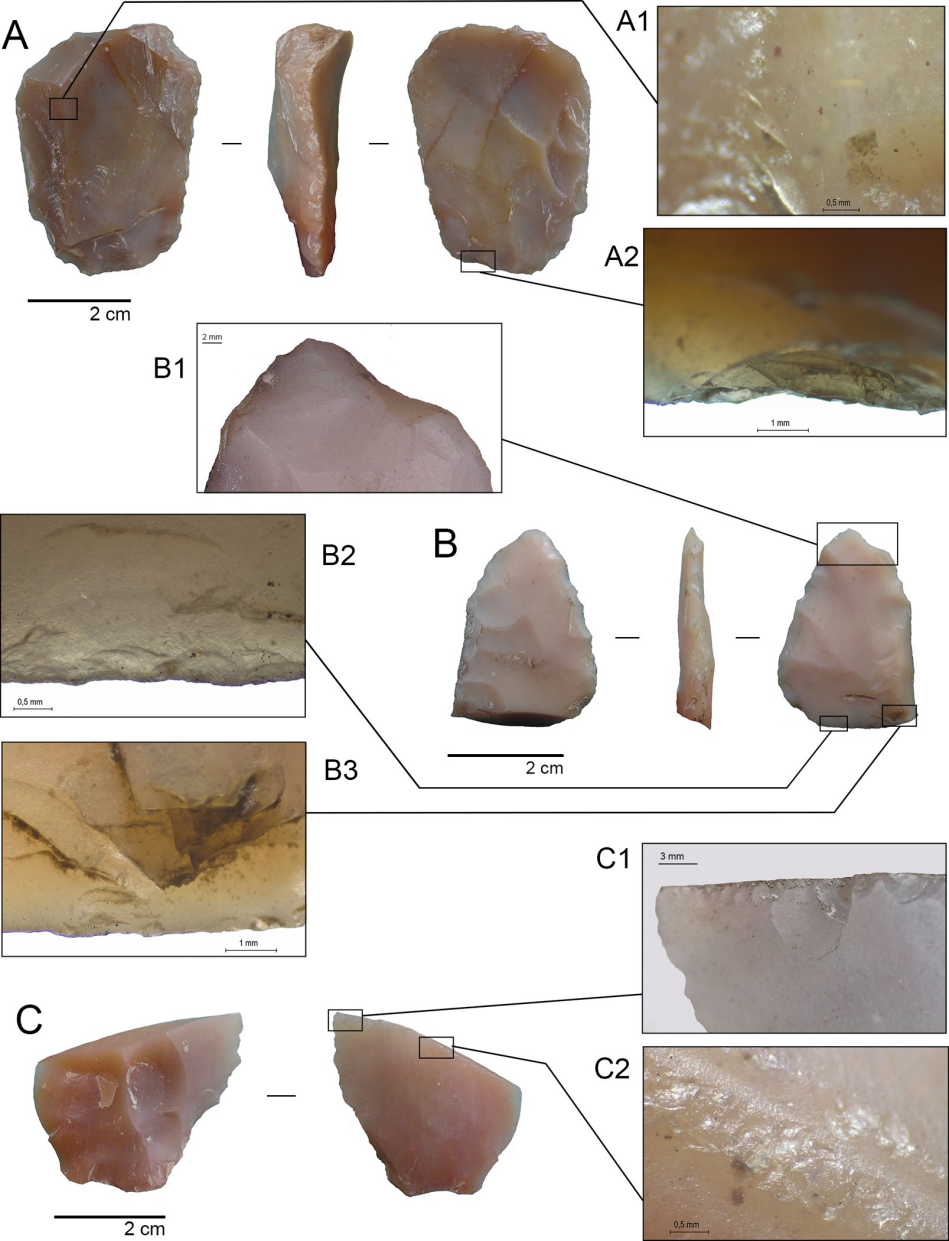
Selected lithic material from TT-3. A. End scraper (TT3-02-N18) in dorsal, lateral, and ventral view; A1. detail of possible red ochre remains next to the dorsal ridge; A2. detail of bending fracture scars with step and hinge termination on the proximal edge; B. Bifacial tool (TT3-01-N18) with a bending fracture on the proximal and lateral portion in dorsal, lateral, and ventral view; B1. detail of an impact scar on the apex; B2. detail of spin off scars derived from a proximal bending fracture, with hinge and step termination; B3. detail of bending fractures’ spin-off scars with snap termination; C. Siliceous flake TT3-G5-N18-03 with thermal alteration cones on the dorsal face and a bending fracture on the distal portion; C1. detail of scarring on the fracture ridge, possibly due to engraving or perforating activities; C2. detail of pitting marks across the bending fracture.

The siliceous rocks are represented by two formal instruments and four pieces of bifacial thinning debitage. The first artifact (TT3-02-N18) is a big end scraper manufactured with a thick greenish yellow blade that shows a dorsal central ridge. Across the technological axis, this tool is 49.4 mm long, 37.6 mm wide and 12.4 mm at its maximum thickness. The working edge of the instrument measures 40 mm in width and the frontal angle from its sliding plane is 61°, showing abundant signs of previous resharpening. During this process, various hinged fractures were formed at the frontal plane of the flaking, which probably led to the piece being discarded. The working edge is tilted to the left, which probably indicates a right-handed user [71–73]. Hafting is suggested for this piece, considering the thinning of the proximal portion and bending fractures with step and hinge terminations located at both the lateral and proximal edges (Fig 7A). There are some possible traces of red ochre on its dorsal surface that require further analysis (Fig 7A1).

The second artifact is a mesiodistal portion of a bifacial tool made from a white siliceous rock (TT3-01-N18). It measures a maximum of 34.9 mm long, 24 mm wide, and 6.3 mm thick. An overshot flaking technique used for thinning this piece was identified, resulting in a very fine tool. Although this technological feature makes the biface more susceptible to breakage, especially if the tool was hafted, it improves its penetration capability [26,74]. One of its lateral edges is broken, as well as the proximal portion, where a bending fracture was identified. In both fractures, abundant spin- off scars with step and snap terminations were recorded, which also produced spin-off scars on the opposite faces [75] (Fig 7B2 and 7B3). A clear impact scar on the apex was also observed (Fig 7B1). All this damage suggests that the biface corresponds to a meso-distal portion of a projectile point broken due to a severe impact [76]. The tool was repeatedly resharpened by bimarginal flaking before its final breakage and discard. However, an inspection under 100x and 200x magnifications only identified scarring and edge rounding, with no striations or diagnostic micropolish from use [77–79], probably due to taphonomic factors.

**Fig 8:**
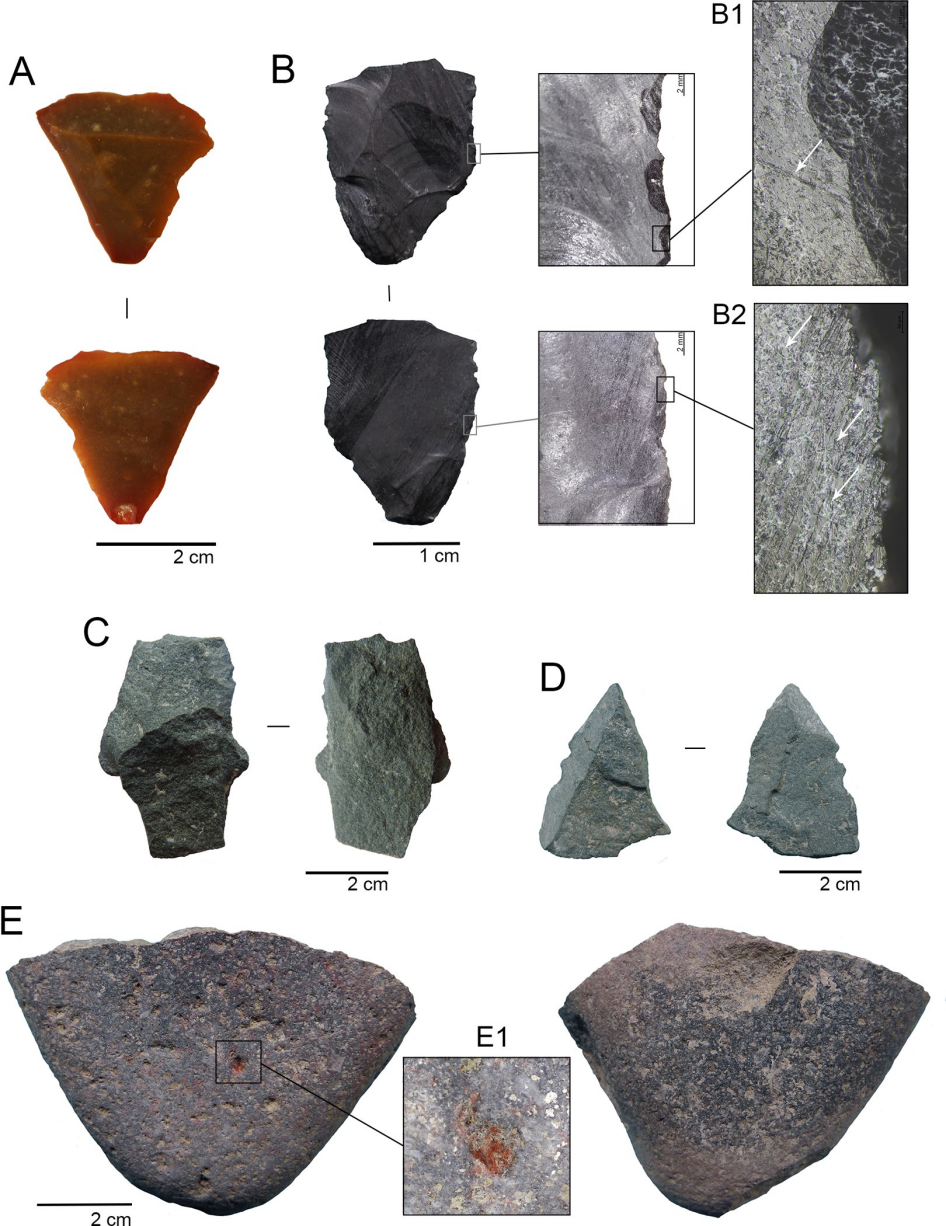
Selected lithic material from TT-3. A. bifacial thinning flake (TT3-D5-N18-10) in dorsal and ventral view; B. Black obsidian flake in dorsal and ventral view; B1. detail of scars with feather termination associated with striation. B2. detail of striations associated with edge damage, parallel and oblique to the edge; C. Middle portion of an andesite debitage flake (TT3-G5-N18-12) in dorsal and ventral view; D. Andesite debitage fragment (TT3-B5-N19-08) with edge damage in dorsal and ventral view; E. Grinding andesite stone tool (TT3-E5-N18-04) with percussion scars, slight polish, and traces of abrasive wear in dorsal and ventral view. E1. detail of red ochre traces.

Siliceous debitage corresponds to two bifacial reduction flakes (TT3-C4-N20-33; TT3-G5-N18-03), one bifacial thinning flake (TT3-D5-N18-10) and one small bifacial retouch flake (TT3-C4-N20-32), none possesses any remaining cortical surface (Figs 7A and 8A). Macroscopically, each flake is different in both color and translucency. The pieces TT3-G5-N18-03 and TT3-C4-N20-33 exhibit marginal continuous flaking on a single edge, with conchoidal and square morphology, also showing feathered or hinged terminations. Both pieces present bending fractures and burination scars on the sharpened fracture’s tip. TT3-G5-N18-03 shows continuous chipping over the fracture at one end, as if it had been used for engraving or perforation (Fig 7C). Two flakes (TT3-G5-N18-03 and TT3-D5-N18-10) show signs of thermoalteration, consisting of clear negative pits or cones (Figs 7C and 8A).

The obsidian bifacial thinning flake (TT3-F6-N18-01) has a broken distal end. It presents continuous non-overlapping and bi-marginal conchoidal flaking at its lateral edges. There are also notable oblique and long, linear markings adjacent to the edges, which, under 100x and 200x magnifications, are consistent with the striae produced by soft material cutting. The surface of the flake has been modified by an unknown chemical interaction, with slight deterioration (Fig 8B).

Coarse grained andesites and basalts are represented by a broken multi-functional ground stone tool with percussion negatives, and seven core debitage fragments (flakes and debris). The ground stone tool (TT3-D5-N18-10) presents a sub-rectangular cross-section, and its general shape might have been ovoidal. The active surface is mainly flat, although slightly concave, showing abrasive wearing and slight polishing, and notably exhibiting remains of red ochre at several discrete points (see below) (Fig 8E). The seven knapping debitage fragments consist of six angular pieces (three of which present cortical surfaces), and a medial flake portion without cortex. Five of the pieces presented non-overlapping scars at their lateral and distal edges, with irregular conchoidal scars from abrupt morphology and step terminations (Fig 8C and 8D). These pieces also presented rounding at one or more edges.

SEM-EDX observation and elemental analysis, together with Raman spectroscopy molecular analysis on TT3-D5-N18-10 was centered inside a small cavity in its active surface (Fig 8E1). Red traces were observed microscopically inside. We concentrated our analysis at this point using SEM-EDX. At first, we observed the presence of Fe associated with Si, Al, Mg, and Ca (S5 Fig). Iron oxide particles are concentrated in the cavity and appear not to be associated with other compounds as observed on elemental mapping obtained using SEM-EDX, indicating the presence of a hematite mineral (S6 Fig). Particles correspond to angular-sided grains of different sizes, between 2 and 5 μm, probably derived from the grinding process. Based on point-group symmetry and a factor group analysis, the optically active normal modes are distributed across the symmetry species of the D^6^_3d_ group [80,81], with 4 active vibrational modes at 228, 298, 410, and 615 cm^-1^, the first one was assigned to A1g, while the other 3 bands were assigned to 3 modes Eg (S7 Fig). With this vibrational analysis, we confirmed the presence of hematite.

#### Combustion feature and archaeobotanical remains

**Fig 9:**
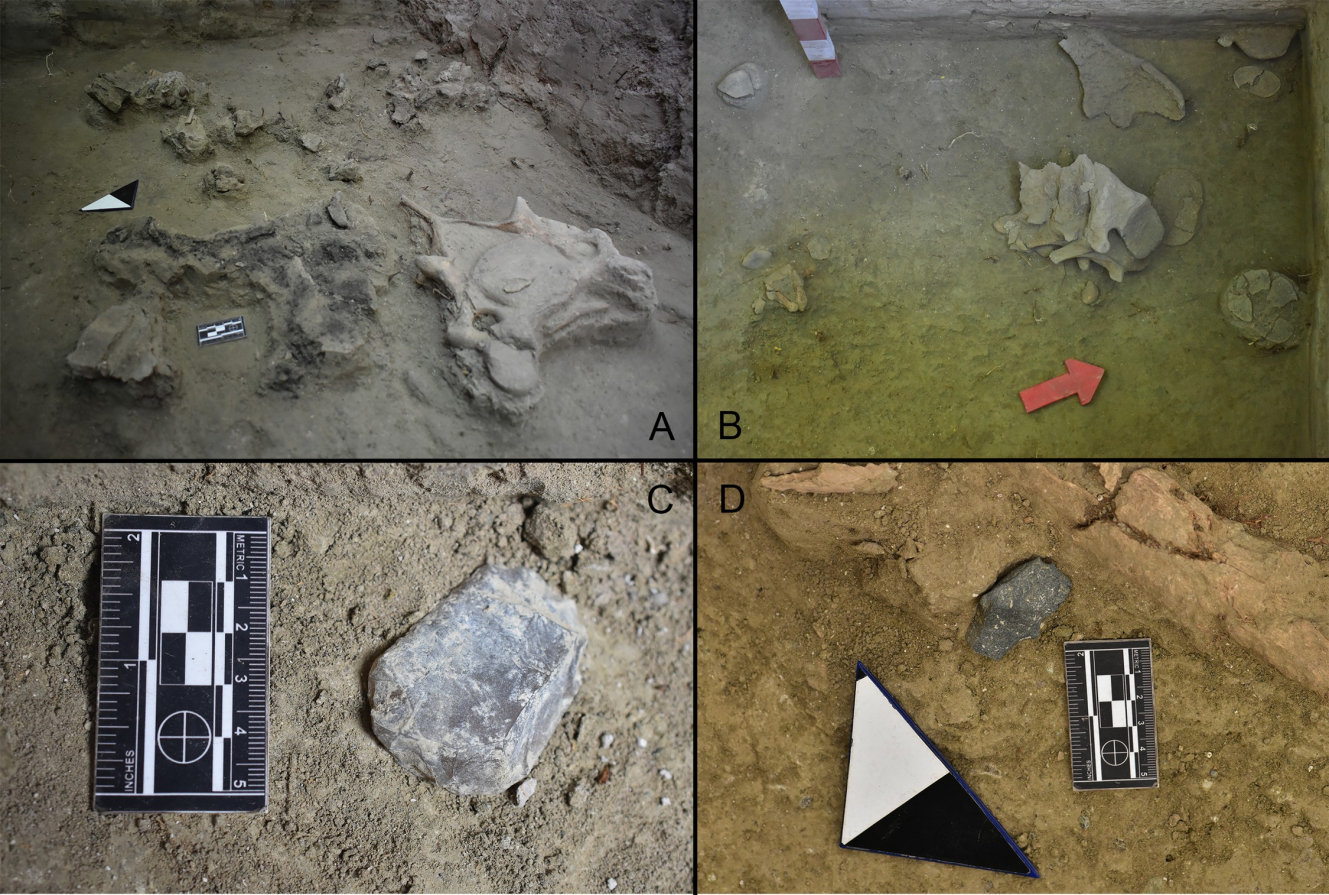
Photographs from TT-3. A. Combustion feature spatially associated with gomphothere cervical vertebrae and skull fragments (excavation unit C5); B. Sacral and caudal vertebrae, vertebral discs, and unfused coxal portions (excavation unit F5). Note the distance between the first sacral vertebra and its vertebral disc; C. End scraper (TT3-U1-N18) *in situ* (excavation unit 1); D. Core debitage (TT3-G5-N18-12) *in situ* spatially associated with gomphothere remains (excavation unit G5).

**Fig 10:**
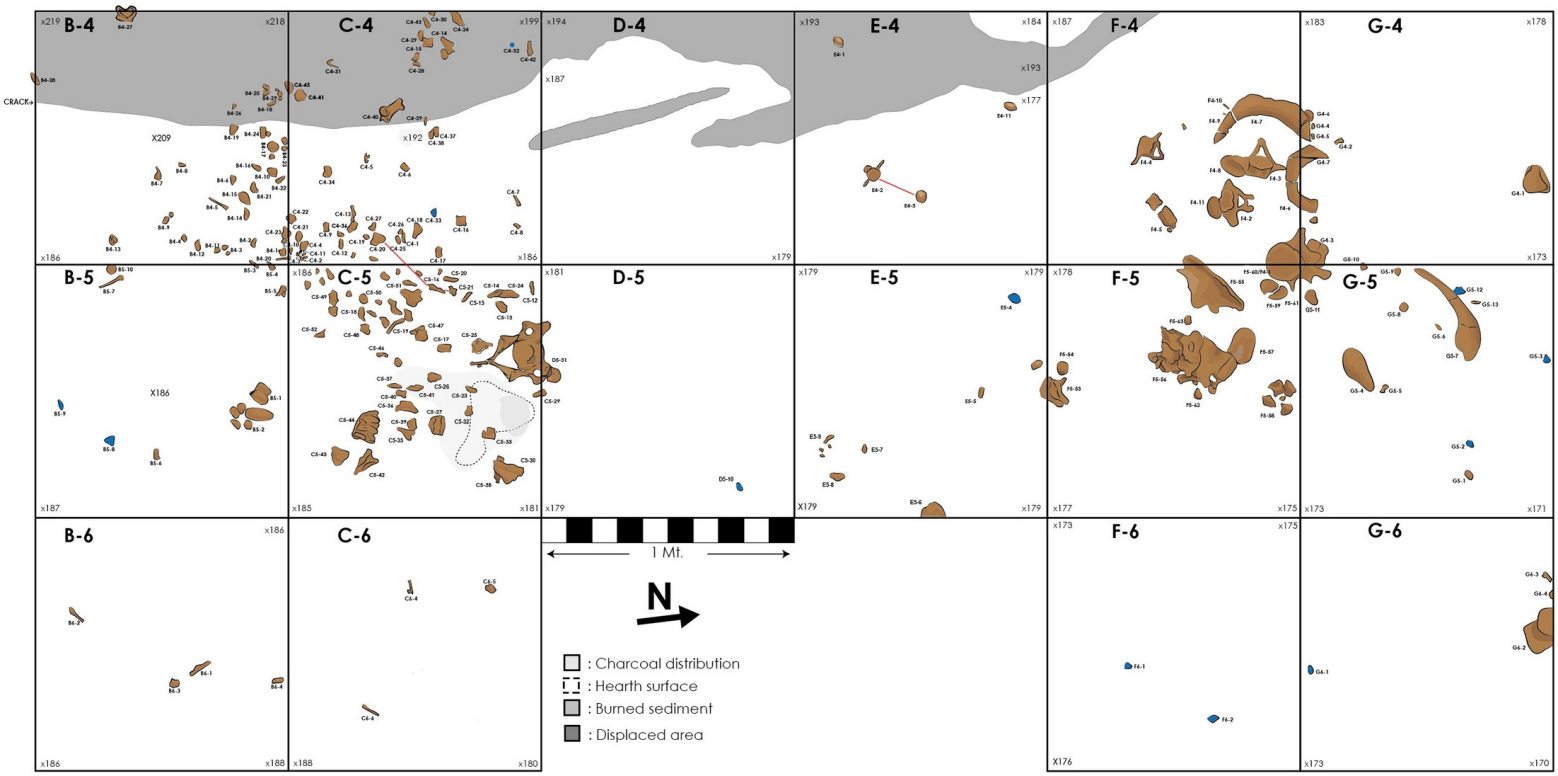
Site plan of the excavated area of TT-3. Light blue objects denote lithic material; brown objects correspond to bone remains; red lines indicate bone refitting.

A combustion feature was recorded *in situ* at excavation unit C5. The hearth begins at the base of L4b, around 177 to 180 cm as a marked carbonaceous area of 24x33 cm (Figs 9A and 10), followed by a small zone of a darkish sediment, probably burnt, measuring 16x11 cm. The feature finished at a depth of 173 cm as a scattering of charcoal spicules and N-S oriented ash, measuring 53x37 cm. Two charcoal samples collected at depths of 175 and 177 cm indicate that the hearth was used between 12,440 and 12,550 cal yr BP (Table 1).

The charred plant remains accounted for 42.5% of the sample studied from the combustion feature. It was possible to identify seeds of *Scirpus* sp (4.7%), those belonging to the cf. Elatinaceae family (4.7%), seeds and fruits of Portulacaceae (22%), seeds of Cactaceae (1.6%), and the remains of an incomplete fruit. Among the non-charred plant remains, a Fabaceae seed, an inflorescence of the genus *Bromus*, an unidentified fruit in poor state of preservation, and abundant remains of seeds and fruits of Portulacaceae were recovered. All of these constitute almost half of the sample (55.1%) (S8 Fig and S9 Table). Considering the large amount of charred and uncharred remains of Portulacaceae, it is possible to suggest a natural deposition of this family. On the other hand, the low proportion of charred remains of Cactaceae, *Scirpus* sp., and cf. Elatinaceae may indicate an anthropic deposition.

The charcoal sample is not well-preserved, preventing detailed taxonomic identification. From a total of 35 fragments, only 15 (42.85%) were positively determined. All the identifiable specimens presented the same anatomic features, being assigned to the Asteraceae family (S9 Fig). Only in some specimens was a slight crushing of the fibers observed, which may indicate the presence of growth rings. All the identifiable charcoal remains presented the same taphonomic modifications (same general aspect and fragility) and belonged to the same taxa, suggesting that the sample analyzed may be associated with a single fire event in which the same Asteraceae woody taxa were burnt.

### Spatial distribution

The site plan clearly shows two main areas where megafaunal remains were documented (south area: excavation units C4, C5 and B4, and north area: excavation units F4, F5, G4, and G5), separated by a virtually fossil-free area (excavation units D4, E4, D5, and E5) (Fig 10). Around the combustion feature, in the south area, the cervical vertebra and a collapsed cranium of the young gomphothere were excavated (Fig 9A). Thousands of its fragments were scattered in a NE–SW direction, possibly indicating the location of its original deposition. The highest number of bone fragments are closer to the hearth. Also, in this concentration, a rib of Artiodactyla undet. and the first phalanx of *Equus* sp. were identified. Two bone refittings were performed, both recovered very close to one other. The major distance recorded was ca. 30 cm (three molar fragments recovered from excavation unit C4 and C5) (Figs 9A, 9B and 10). In the north area, several elements of the caudal part of the column were identified, some of them articulated. The bones include four unfused sacral vertebrae, three of them articulated (Fig 9B), a lumbar vertebra and four caudal vertebrae. Spatially associated with the latter, many fragments of unfused tuberosities and ridges of the pelvis were recovered. In some cases, the vertebral discs were in close proximity to their respective vertebrae. In this regard, the largest distance between a vertebra and its disc was approximately 15 cm (Fig 10). Lumbar discs detected in the wall of the excavation unit F6 suggest that bone deposition continues farther to the north. Finally, the sesamoid, tarsal, and carpal bones, which have no anatomical coherence with the vertebral concentration, were recovered on the north side but outside this main concentration.

**Fig 11:**
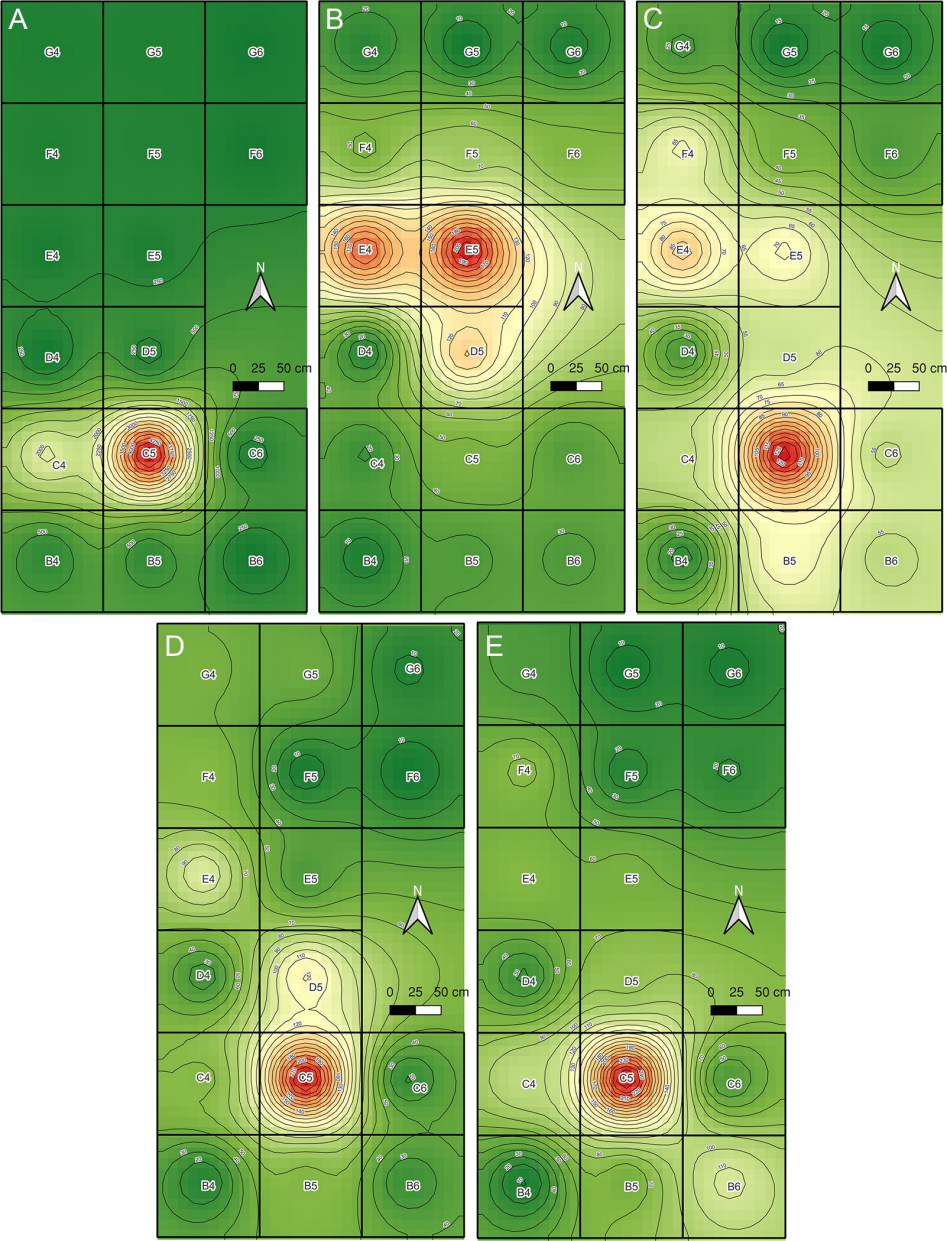
Heat maps of faunal remains distribution at the lower level of facies L4b. A. Megafauna; B. Rodents; C. Birds; D. Amphibians; E. Fish.

Considering all the specimens recovered from the lower level of facies L4b, we constructed heat maps segmented into general taxonomic categories to observe the faunal distribution. There is a marked concentration of megafaunal and small vertebrate specimens in excavation unit C5. The only exception corresponds to rodents, which are scarce in C5 and more abundant in excavation units E4 and E5 (Fig 11). These results are consistent with the location of the anthropogenic hearth. Gomphothere bones located in the north area are not observed in the heat map, due to the large amount of small skull fragments recovered from excavation unit C5.

On the other hand, lithic material was recovered scattered around the excavated area without configuring any concentrations. In fact, except for pieces TT3-G5-N17-12, TT3-C4-N17-32, and TT3-C4-N17-33 (Figs 9C, 9D and 10), all the lithic artifacts were recovered relatively close to, but not directly associated with megafaunal remains. Also, there was no strict spatial relationship between the combustion feature and the lithic material, although they do exhibit unequivocal fire exposure markings. All the lithics were recovered in a horizontal position.

## Discussion

### Formation process, taphonomy and context integrity

Several lines of evidence indicate a mostly undisturbed context in the lower portion of facies L4b, discarding major vertical migration and/or horizontal displacement of artifacts and megafaunal remains prior to and after their burial. The sedimentary sequence is composed mostly of a succession of fine-grained sediments, mainly silts of lacustrine origin without reworking, suggesting that it was generated due to a continuous sediment input without significant truncations. The age-depth model is fully coherent with this sedimentological scenario since it is internally consistent with no major reversal in radiocarbon dates or hiatuses throughout most of the Holocene sequence. The age estimate for the base of the L4b facies is also consistent with two independent radiocarbon dates obtained on the charcoal from the combustion feature.

Five sterile facies (L3b, L3c, L3d al L3e, and L4a ca. 40 cm) provide a boundary between the Late Pleistocene anthropogenic deposit (facies L4b) and the following human occupation in facies L2 (∼ 8,200 cal yr BP). No megafauna remains have been found in Holocene facies. This is consistent with the absence of dung-fungus *Sporormiella* sp., which has been detected only in facies L4b [53]. Also, the lithic assemblage of these Holocene hunter-gatherers is entirely technologically different from those located in facies L4b [82], thus any vertical migration would have been easily detected, which is not the case. Although we observed some bioturbation (mainly burrowing activities and uprooting), especially in the western part of the excavation site, this only affected the Holocene facies (mainly L1 and L2) and did not extend into the Pleistocene sediments.

Larger horizontal displacements can be discarded as well. Facies L4b was deposited horizontally across the whole excavated area, and only a minor orientation toward the center of the lake was observed. Moreover, the very fine sediments that constitute the facies suggest a low-energy depositional scenario, incapable of transporting megafaunal bones or artifacts [83]. This interpretation is sustained by the spatial and contextual features recorded at the site. Firstly, an *in-situ* combustion feature was excavated at the lower level, which is spatially associated with small-sized bone fragments. In a fluvial or colluvial scenario, this feature and the surrounding small bones would have been washed away. The same is true for several of the complete gomphothere bones recovered, such as sacral, caudal vertebrae and astragalus, all of them with high Fluvial Transport Index values (>80) according to [84]. These categories also concur with Voorhies Group 1 (easily transportable) [85]. Moreover, we found two concentrations of gomphothere bones, separated by an almost sterile area of 4m^2^. Lithic material was recovered scattered around the excavated area and was recorded always in horizontal positions. Finally, various unfused vertebral discs were recovered in proximity to their corresponding vertebrae, indicating almost no displacement after the decay of their soft tissues. In this regard, the bone refitting includes cranial bones and dental pieces from the same excavation unit or adjacent units. The post-depositional landslides detected in the west area only generated an intra-facies displacement, meaning that some fossil remains were recorded at greater depths than the rest of the undisturbed remains (Fig 9 and S2 File).

Taphonomically, megafauna bones do not show signs of having been abraded (which would suggest water transport), weathering is absent, and carnivore damage is especially low, indicating that big scavengers were not important in the assemblage conformation or displacement. [86] observed two carcasses of elephants, less than two months after having been butchered by the Bisa people in Zambia, and recorded a relatively high proportion of gnawed bones (41.6 and 36.2%). The fact that tooth marks were detected in a low-meat anatomical element suggest that the scavengers had late access to the carcasses [87], probably after anthropogenic processing. In contrast, the most common taphonomic modifications are root etching and manganese coating, which occurred after the remains had been buried or underwater. Thus, this evidence coupled with low frequencies of other taphonomic modifications capable of displacing skeletal elements, such as trampling, strongly suggests that the spatial distribution of megafaunal remains and lithic artifacts in the lower level of L4b reflects mainly the human activities that occurred at the site. In addition, it confirms a relatively rapid burial of the assemblage.

On the other hand, the small vertebrate assemblage indicates a more intricate taphonomic history. According to the taxonomic diversity, its comparative frequencies, and the differential presence of cutmarks, fire marks and gastric corrosion (S3 File), we interpret that at least a portion of these subsets in the lower level of facies L4b was anthropically introduced. Rodents and birds would have been more anthropically exploited than anurans and fish if the proportion of fire marks are taken into account. Low intensity of gastric corrosion is indicative of a small contribution by nocturnal raptors to the assemblage via pellets. Only in the anurans’ subset, carnivores and/or diurnal raptors (i.e., falconids) could have contributed as well, due to more intensive gastric corrosion evidence. Natural modification of fish remains indicate a deposition mainly via natural deaths (S3 File). Finally, the incorporation of eggshells into the assemblage could be either natural or anthropogenic, since some of the most common anatid species in central Chile (*Anas georgica*, *Anas flavirostris*) build their nests on solid ground, trees, or rocks, but near to shore [88], coincident with the location of the TT-3 Pleistocene occupation.

### TT-3: Site function, chronology, and its relationship with other contemporary sites

Artifact and faunal remains, as well as their spatial distribution within TT-3, are indicative of a possible hunting event and the primary butchering of a single young gomphothere. Hunter-gatherers also processed and consumed small lacustrine fauna, fruits, and other plant portions. Other activities were carried out as well, such as ochre grinding and bone/hide preparation. New paleoenvironmental and paleoclimate reconstruction, considering isotopic data from the gomphothere recovered at the site (S4 File), indicate that these events occurred under warm (Mediterranean) conditions. The differences between these results and previous studies (i.e., [44]) could be related to differences in the type and temporal scale of the proxies employed.

Ethnographic data on elephant hunting is consistent in pointing out that the butchering process occurs in the same place where the animal died, considering the great weight of the carcass and the difficulty in handling it [89–93]. Moreover, the gomphothere’s head was probably the heaviest segment of the carcass if the data on modern adult elephants are taken into account (ca. 436 kg, [94]). So, it is reasonable to conclude that, in the case of TT-3, all butchering activities were performed around this anatomical element. Prey size usually determines the butchering techniques and which segments of the carcass are taken away to a residential camp [95–99]. Observations made of the Hadza in northern Tanzania indicate that animals of size IV-V (400–2000 kg) are comparatively more likely to be butchered at the kill site than any other size classes [97,98,100]. Consequently, complete, or nearly complete, de-fleshed carcasses (butchering mode I according to [97]) are expected to be left at the kill/processing site. In six of eleven Hadza giraffe (*Giraffa camelopardalis)* processing locations [97], no bones had been transported to the residential site, other than a few ribs. Distance from the residential camp is also related to prey size, since bigger taxa (which are less numerous on the landscape) are usually hunted (or scavenged) farther from the base camp than medium/small prey [100].

Nevertheless, an elephant (or a gomphothere) is two or three times bigger than a giraffe. Ethnographic data on the Mbuti pygmy from the Ituri forest demonstrate that a unique settlement pattern is configured when an elephant is hunted (or a carcass is found), which does not fit strictly within the traditional base camp/task camp dichotomy. Normally, all the people living at the residential camp, but also other related groups not directly involved in the hunt, move to the kill/encounter site, creating a temporary camp surrounding the dead proboscidean [89,92,93,101]. [86] observed that the butchering process occurs in an area of ca. 225 m^2^, and [90] reports an average of 444m^2^ for the entire temporary camp, with at least 10 m between the carcass and the nearest residential structure. Elephants are completely de-fleshed, especially on account of several butchering parties, and the stripped meat is transported to the temporary camp for consumption, but also to be dried to prolong its edibility [89,92]. The butchering process takes a few hours, but the activity around the carcass can extend to over a week, because other significant activities are also performed (feasts, transmission of information, marriages, and so on; [89,101,102]. Consequently, we believe that the evidence excavated at TT-3 mainly reflects the logistic area of a large settlement in which both residential and butchering areas were spatially differentiated.

The spatial distribution of megafauna bones forming at least two separated clusters indicates that the disarticulation of some segments had occurred, and that these had likely been subject to further processing. The absence of limb bones and other anatomical units at TT-3 is probably a stochastic effect related to the small area excavated. However, we cannot rule out the possibility that some bones had been taken away by big carnivores, considering the astragalus with tooth marks. Heat maps suggest a marked concentration of small vertebrates around the anthropogenic hearth, indicating that some of these taxa were consumed near to this feature. Burned gomphothere bones in close association with the anthropogenic hearth indicate that both depositions were synchronic. This interpretation is reaffirmed by two radiocarbon dates, and the presence of only one taxon in the charcoal remains, all of them with the same taphonomic attributes. All these features confirm a main single depositional event. However, the identification of a rodent bone cluster in excavation units E4 and E5, some of them bearing fire marks, could indicate another consumption event not necessarily related to gomphothere processing.

Cutmarks on caudal vertebrae are a clear marker for anthropogenic manipulation. According to Haynes and Krasinski’s revision (2021) [103], cutmarks on proboscidean vertebrae from archaeological sites are rare. [104] describe a caudal vertebra with cutmarks on its body from the Pavlov I site (Late Pleistocene) that was interpreted as having been produced by skinning activities, due to the absence of meat at this place. Ethnographic studies of Pygmies from the Ituri forest in Africa indicate that the trunk and tail of the dead elephant are cut off as trophies. They symbolize the entire animal, given that they are the parts that lead and trail respectively [89,102]. Moreover, the tail has thick hairs that are used to make bracelets [89].

Ethnographic information on elephant butchering/temporary camp sites indicates that vegetables are also regularly employed during their occupation in both domestic and ritualistic activities [89], therefore, presence of cacti and *Scirpus* sp. seeds at TT-3 are not surprising. Both taxa hold robust ethnographic significance within the studied region, encompassing diverse contexts such as food, construction, and medicinal uses [105,106]. Although we are just beginning to assess the anthropogenic use of vegetables at the site, we believe that they were certainly used systematically for several purposes. This is true especially if we consider the large number of taxa (24 of a total of 53, 45.2%) with traditionally known uses (medicinal, food, basketry, dyeing) described at the lake prior to its desiccation [107]. In this respect, archaeobotanical studies undertaken at other contemporary late Pleistocene sites in Chile, such as Mani 12 and 32 in the northern area, or Monte Verde II in the Patagonian Forest proved relevant to the discussion of the role of plants in early hunter-gatherers’ lives [108–111].

Technological, morpho-functional, and preliminary use-wear analysis of the knapped lithic assemblage is consistent with hunting, cutting on soft materials, scraping, and piercing/engraving activities associated to gomphothere and small vertebrate butchering, but also to hide and/or bone preparation. Nor can plant processing be ruled out. If we consider the quality of the raw materials and their provenance, there is a clear twofold strategy. The exotic and possibly exotic high-quality raw materials (siliceous rocks and obsidian), which constitute ca. 50% of the assemblage, shows curated technology features: formal, long-lived, hafted tools with intense edge resharpening until they are discarded; bifacial flakes (with no bifacial cores recovered) from the final stages of reduction without cortex used as cutting tools, and the presence of one small piece of retouch debris indicative of the manufacturing/resharpening of an instrument not recovered at the site. On the other hand, local raw materials (coarse grained basalts/andesites) were used as expeditive tools without a formal formatting treatment, some of them still exhibiting cortex from the original nodule.

The possible presence of a fractured mesiodistal portion of a projectile point (TT3-01-N18) suggests hunting activities. The clear bending fractures on its proximal and lateral portions, in addition to the impact scar on the apex, indicate a fracture while the piece was still hafted [76]. Interestingly, the bifacial thinning technique observed includes overshot or edge-to-edge flaking, a feature observed in Fishtail Projectile Points (FPPs) [112,113]. Also, its thickness (6.3 mm) is similar to the measures obtained from the two FPPs recovered from TT-2 (5.8 mm and 6.4 mm, [35]) and also from a FTPP stem (5 mm) collected in the Santa Inés locality, situated only 2 km from TT-3 [114]. This is also consistent with the average thickness of the FPP studied by [26] from several localities in southern South America. According to these authors, the thinness of the FPP design would cause a bigger wound to large game. In addition, breakage experiments conducted with FPP replicas indicate that most of the bending fractures are located transversely across the stem, forward from the narrowest portion, which is coherent with the location of the fracture in the TT-3 biface [76]. Although we were unable to recover the stem, which is the most diagnostic portion of the FPP, the technological features identified, as well as the location of its fracture, point to a broken and discarded FPP [26,76,112,113]. The dates obtained from TT-3 fall well within the Fishtail Projectile Point horizon in South America between 13,000 and 10,900 cal yr BP [26].

Unretouched flakes seem to have been employed in TT-3 carcass processing. These kinds of tools have been tested in several elephant butchering experiments with dissimilar results. [115,116] argue that unmodified flakes can be used satisfactorily either for skin removal or meat stripping. However, [117] indicates that such instruments are not suitable for the former action. Recently, [118] have highlighted the importance of bifacial technology in their actualistic butchering experiments, especially if the instruments are hafted. The absence of bifacial technology for butchering in the site is not surprising, considering its almost non-existence in the toolkit described for TT-1 and TT-2. Moreover, in the latter site, unretouched and retouched blades were apparently used for this purpose [35]. It must be kept in mind that the logistical condition of the site and the curated technology recovered would prevent the discarding of long-lived tools. In the same way, there are clear, intense and different wear traces on three of five unretouched flakes of exotic and possibly exotic raw materials in TT-3, In fact, two siliceous flakes were intentionally used over their fractured edges, a technological feature very similar to "radial break tools’’ described by [119] for some Folsom Late Pleistocene sites in North Dakota.

Lithic debitage is surprisingly scarce in TT-3. Nevertheless, elephant butchering experiments (i.e. [118]) suggests that the resharpening of lithic instruments it’s a regular task during the carcass reduction process. Discarding a sampling problem since all the sediments were wet-sieved using 2 mm screen meshes (S1 File), we hypothesize that resharpening activities were carried out outside the excavated area.

In the lithic assemblage, it’s remarkable the presence of a fractured cobble with red ochre remains on its abraded surface, indicating its use for grinding iron oxide (hematite). Pigment has been recorded at Late Pleistocene archaeological sites in both the Americas, either in task camps, residential camps, funerary, and other non-utilitarian contexts [120–124], thus occupying an important role in several aspects of hunter-gatherer life. In domestic contexts it has been associated with leather preparation [67], but it could also be used for hafting processes, artifact decoration, or even body painting, as archaeological and ethnographic data suggests (i.e., [121,125,126]). Several of these activities could have been carried out at TT-3.

Regarding seasonality, in central Chile the flowering and fruiting season of many species of Cactaceae occurs in the spring and summer months, respectively [127–129] and many aquatic birds begin laying their eggs during early spring [88]. Although there is scarce record of charred Cactaceae seeds and anatid eggshells in the lower portion of L4b, these evidence are sufficient to suggest that the activities carried out at TT-3 occurred during the dry season.

Two radiocarbon dates obtained from isolated charcoals in facies L4b are statistically similar (p <0.05) to those obtained in the combustion feature and probably correspond to the same occupational event. A third date is significantly different from the former group (ca. 12,680 cal yr BP) but is similar to the recently published dates of TT-1 [33]. These results suggest a recurrent occupation of the ATTL during the 12,600–12,400 cal yr BP span.

The relative chronological synchrony between TT-1 and TT-3, the fact that they were deposited in the same sedimentary context, and their proximity (ca. 40 m) allows us to discuss both sites from a local perspective. TT-1 was interpreted as a small camp considering the lithic assemblage [35], but despite the large surface excavated (ca. 189 m^2^, [31,32] only 88 lithic specimens were recovered (2.14 lithic/m^2^). Most of them are debitage and core flakes [35,130] which are nearly absent from TT-3. In the latter, 15 lithic artifacts have been recovered in 18 m^2^ excavated (1.2 lithic/m^2^). Nevertheless, at both sites there is a mixture of fine-grained and medium/coarse raw materials, and, regarding the former, siliceous rocks and obsidian were both identified as long-lived formal tools. In contrast to TT-3, Canidae, Cervidae, and Equidae are well represented at TT-1 [33]. Therefore, if the gomphothere remains are also considered, the mammalian record of TT-1 is comparatively more diverse. In addition, the anthropogenic imprint on the small vertebrate assemblage seems to be more important at TT-1 than at TT-3 [34]. Gomphothere skull portions as well as mandibles and pelvises were recorded at TT-1 [33], indicating that the primary butchering process was carried out around these anatomical units. At both TT-1 and TT-3, no tusks were recovered. Considering all these features, we propose that the faunal and lithic record of TT-1 is compatible with one or more megamammal butchering/temporary camps where there is a low discard of formal artifacts made from fine-grained raw materials. Therefore, we sustain that TT-1 and TT-3 are functionally the same type of site, distinct only in that TT-1 was more extensively excavated, and thus the more residential areas of the former site were exposed. Considering their temporary condition, more stable residential camps would have been located elsewhere, even outside the lake basin. The absence of gomphothere tusks in both sites could be explained as transported from these anatomical units to the more permanent residential camps.

During Late Pleistocene times, due to the predictable and readily available animal and plant resources, the shore of the ancient Tagua Tagua lake acted mainly as a hunting/scavenging ground. Several highly mobile bands would have met on a regular basis, generating spatially separated but functionally similar temporary camps (see also [131]). Ethnographic data suggests that elephant hunting (or scavenging) was not necessarily a communal task. However, the resulting vast amount of meat was the perfect excuse to perform many other activities oriented to generate or reinforce social relations and the sense of belonging among related bands. In Pleistocene times, when a low population density is assumed, these ventures and their recurrence were probably crucial to ensuring the success of the groups that inhabited the area on a regional scale.

Considering the dates obtained from TT-3 and TT-1, this mobility and settlement strategy was well established as early as ∼12,600 cal yr BP and probably continued until the until the very last part of the Pleistocene and the Early Holocene (∼11,600 cal yr BP), as the evidence of TT-2 seems to suggest ([32], but see [35]). In fact, the significative exploitation of gomphotheres observed in TT-2 would indicate a progressive increase in the use of the basin, probably associated with the rapid and intense climate changes that occurred during the Pleistocene-Holocene transition [44,53].

At a more regional level, the presence of obsidian at TT-1 and TT-3, along with possibly siliceous rocks, suggests that the lake was a component of a more intricate and extensive mobility circuit that encompassed at least the Andes Mountain range. This suggests a comprehensive understanding of the regional landscape and its resources. Specifically, in the case of TT-3, these movements could extend ca. 100 km, as XRF analysis points out. Crystal quartz is another high-quality rock that presumably circulated long distances in the central and semiarid region of Chile during the terminal Pleistocene [35,132]. Although the crystal quartz quarry workshop of Valiente, located ca. 300 km to the north, is chronologically contemporaneous with TT-1 and TT-3 [132], there is no record of such rock at these sites. In fact, quartz is only recorded 1000 years later at TT-2, where FPP made of this raw material have been found, but where there is also a total absence of obsidian [32,130]. Even if the quartz recorded in TT-2 has no relation to the Valiente quarry, these features suggest that the lithic procurement behavior changed in the lake area over this period. And thus, it is also indicative of variability in the use of the space, mobility, and/or the interaction of these early hunter-gatherers in the rapid-changing landscape of the late Pleistocene.

- On a subcontinental scale, Late Pleistocene open-air archaeological sites with clear and undisturbed human-megafauna interaction are relatively rare (see [133] and references therein). Moreover, human-proboscidean interaction is even rarer, with no more than seven sites, most of which have been interpreted as killing/butchering sites (Taima-Taima, Tibitó, El Cautivo, Monte Verde II, TT-1, TT-2, and TT-3; [24,31,32,134–136]). Chronologically, both Monte Verde II and Taima Taima are dated to before 14 ka cal BP and coincidentally both contain El Jobo projectile points [24,135]. The remaining sites are younger, ranging from about 13.5 to 11 ka cal BP, but only TT-2 and possibly TT-3 have yielded FPP [91]. Interestingly, most of these well-recognized contexts are located near freshwater sources, creating a pattern that could certainly guide future research. Indeed, this pattern can be extrapolated to other contemporary South American Late Pleistocene archaeological deposits with different megamammal records, such as Campo Laborde [137].

## Conclusions

Considering the evidence discussed previously, TT-3 can be interpreted mainly as a single gomphothere hunting and processing event that occurred during the dry season, inserted within a relatively undisturbed sedimentary context. Other activities were carried out, such as plant and small vertebrate processing/consumption, as well as ochre grinding. The detection of a wide spectrum of activities is interpreted here due to the mixture of logistic and domestic tasks that probably occurred at the site. The chronological and contextual data indicates a close relationship with the nearby TT-1 site, which in turn can be linked to a recurrent occupation of the lake’s shore during a period that spanned ∼12,600–12,400 cal yr BP.

Behavioral Ecology posits that, after a colonization phase, hunter-gatherers would always settle in the more suitable habitat first (i.e., considering the impact potential on the individual’s evolutionary fitness). Thus, these areas will always have a higher population density [138]. Even though it is difficult to evaluate quantitatively how suitable an extinct habitat was, the evidence discussed here, as well as the information from contemporary sites, allow us to interpret that, during the terminal Pleistocene, the ATTL was probably one of these central localities or nodes. This assumption rests not only on an economic view (i.e., diversity of taxa exploited) but also on the social implications of the inferred settlement pattern, if the ethnographic information is taken into account.

The relatively high presence of anthropogenic evidence in a small area of ∼0.04 km^2^ would support our conclusions; however, if the total lake surface is considered (∼30 km^2^), new non-invasive and invasive surveys are needed to find other contemporaneous or even earlier archaeological deposits, and thus evaluate some local-scale issues previously discussed. The fine-grained strategy we pursued, favored by the exceptional human record and preservation conditions at the ATTL will certainly improve our knowledge of these early human adaptations to highly attractive extinct habitats. Therefore, it will help to construct a more robust and comprehensive picture of the late Pleistocene hunter-gatherers of southern South America.

## Supporting information

S1 Fig East profile of units F6 and G6. (PDF)

S2 Fig *Equus* sp. first phalanx heavily coated with MnO2 in anterior view. (PDF)

S3 Fig Eggshells. Top: *Spatula clypeata* actual eggshell. Bottom: Archaeological eggshell fragment assigned to Anatidae undet. from archaeological unit D5, Level 18, Layer L4b.
(PDF)

S4 Fig Scatterplot of Sr/Rb versus Rb/Zr for obsidian artifacts from Mendoza and Central Chile. Source grouping follows Barbarena et al. (2019) and Sanhueza et al. (2022). Values obtained for artifact TT-F6-N18-01: Sr/Rb: 0.518; Rb/Zr: 0.473. Values obtained for Laguna El Maule samples: Sr/Rb: 0.526, 0.519, 0.537; Rb/Zr: 0,757; 0,804; 0,772, respectively.
(PDF)

S5 Fig SEM-EDX spectra of red particles inside a cavity of the lithic artefact. (PDF)

S6 Fig Mapping image of inside and a border of a cavity on lithic artefact. In red, the presence of iron oxide is more abundant inside the cavity than on the border.
(PDF)

S7 Fig Raman spectra of red mineral particles present in the cavity of a lithic artefact. (PDF)

S8 Fig Plant remains from TT-3 combustion feature. a) *Scirpus* charred seeds. b) seed of cf. Elatinaceae c) Charred Portulacaceae fruit. d) Uncharted Portulacaceae seed and fruit. e) Cactaceae seed. f) Unidentifiable fruit. g) Fabaceae seed. h) *Bromus* sp. Inflorescence. i) Unidentified fruit.
(PDF)

S9 Fig Transversal plane (180x and 300x) of charcoal fragments assigned to Asteraceae from the combustion feature of TT-3. (PDF)

S1 Table Frequencies of gomphothere skeletal parts. (XLSX)

S2 Table Frequencies of rodent skeletal parts. (XLSX)

S3 Table Frequencies of bird skeletal parts. (XLSX)

S4 Table Frequencies of anuran skeletal parts. (XLSX)

S5 Table Frequencies of fish skeletal parts. (XLSX)

S6 Table Frequencies of taphonomic modification on small vertebrates, excavation units C5, B4, and B5 (NISP). (XLSX)

S7 Table Intensity of MnO2 coating (NISP). (XLSX)

S8 Table Intensity of gastric corrosion (NISP). (XLSX)

S9 Table Summary of plant remains and their condition. (XLSX)

S1 File Material and methods. (PDF)

S2 File Description of post-depositional alterations on the west area of the TT-3 site. (PDF)

S3 File Origin of the small vertebrate assemblage at TT-3 site. (PDF)

S4 File Stable isotopes analysis from gomphothere tooth (bioapatite) from TT-3 site. (PDF)
