# Correction: Taguatagua 3: A new late Pleistocene settlement in a highly suitable lacustrine habitat in central Chile (34°S)

**DOI:** 10.1371/journal.pone.0306861

**Published:** 2024-07-05

**Authors:** Rafael Labarca, Matías Frugone-Álvarez, Liz Vilches, José Francisco Blanco, Ángela Peñaloza, Carolina Godoy-Aguirre, Álvaro Lizama-Catalán, Cristóbal Oyarzo, Carlos Tornero, Erwin González-Guarda, Ayelen Delgado, Marcela Sepúlveda, Paula Soto-Huenchuman

The images for Figs 4 and 5 are incorrectly switched. The image that appears as Fig 4 should be Fig 5, and the image that appears as Fig 5 should be Fig 4. The figure captions appear in the correct order. Please see the correct Figs 4 and 5 here:

**Fig 4:**
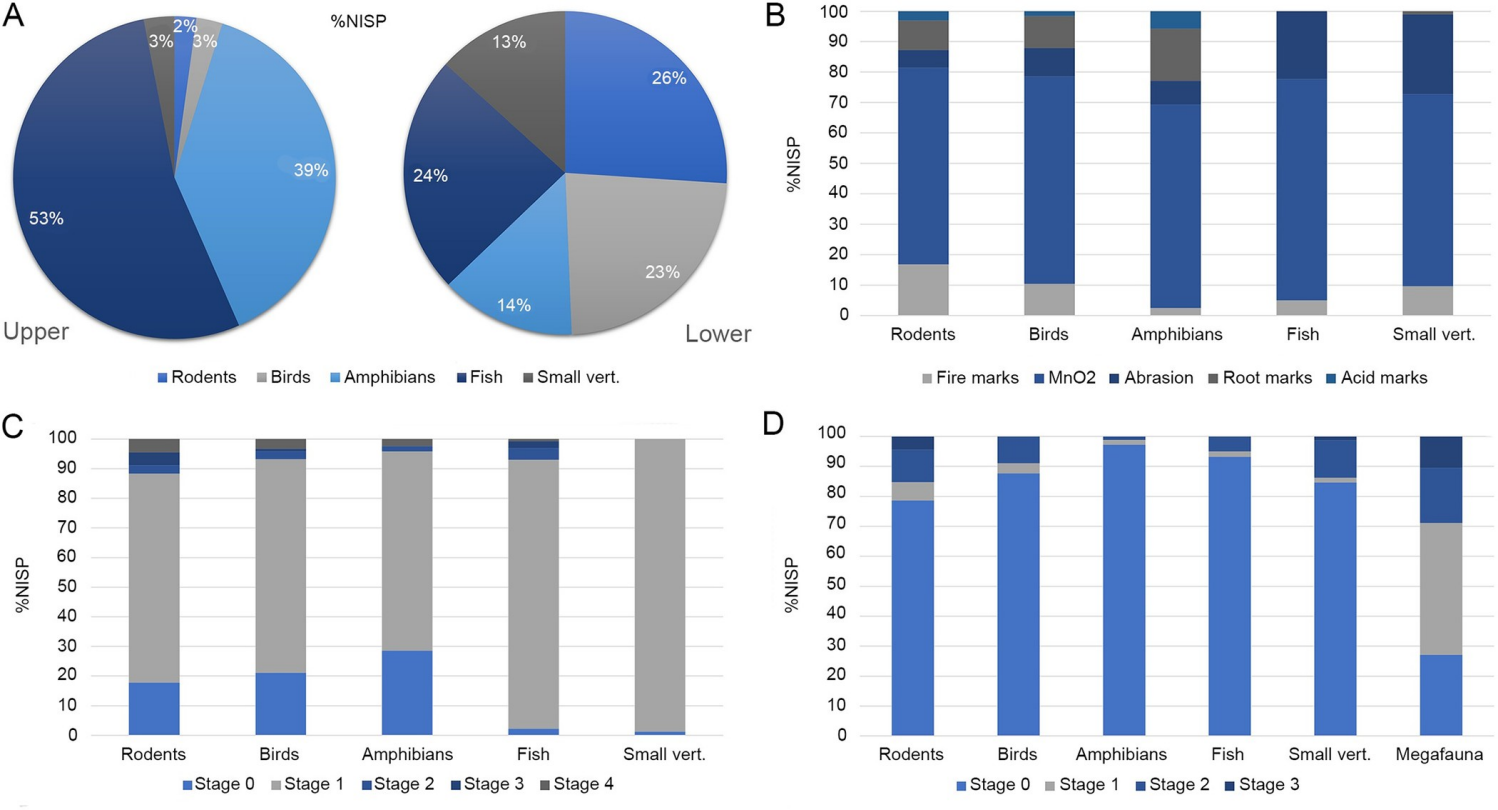
Relative frequencies of faunal remains and taphonomic modifications. A. Small vertebrate distribution on facies L4b; B. Taphonomic modifications on the lower level of facies L4b. C. Manganese intensity on the lower level of facies L4b; D. Fire intensity on the lower level of facies L4b. Manganese intensity was recorded as follows: Stage 0: unmodified; Stage 1: Light (<25% of the surface covered); Stage 2: Moderate (<50% of the surface covered); Stage 4: Severe <75% of the surface covered); Stage 5: Extreme (100% of the surface covered). Chromatic stages of fire action were recorded as follows: Stage 0: unmodified; Stage 1: mostly brown; Stage 2: mostly black; Stage 3: mostly gray/white.

**Fig 5:**
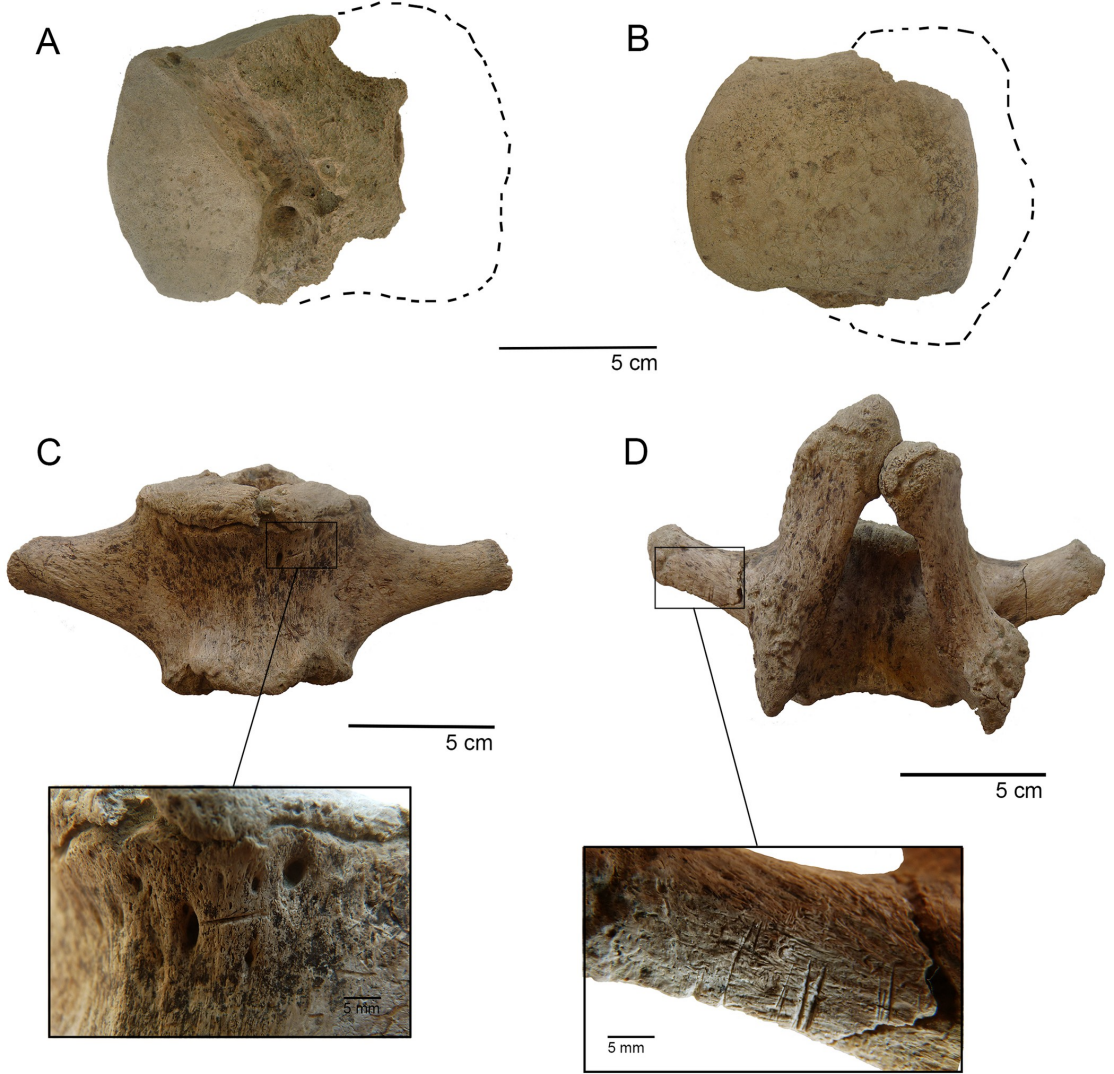
Taphonomic modification on gomphothere bones. A-B. Astragalus (TT3-E5-N18-06) with tooth marks in ventral and dorsal view (discontinued line indicates the original extension of the specimen); C. caudal vertebra (TT3-E4-N18/19-02) with a single cutmark; D. caudal vertebra (TT3-E5/F5-N18-53) with several parallel cutmarks.
