# Supplementary material for: Taguatagua 3: A new late Pleistocene settlement in a highly suitable lacustrine habitat in central Chile (34°S)
Source: PLoS One. 2024 May 22;19(5):e0302465. doi: 10.1371/journal.pone.0302465 (PMC11111044; doi:10.1371/journal.pone.0302465)
Supplement: S3 Fig — Top: Spatula clypeata actual eggshell. Bottom: Archaeological eggshell fragment assigned to Anatidae undet. from archaeological unit D5, Level 18, Layer L4b. (PDF) [file pone.0302465.s003.pdf]

Taguatagua 3: a new late Pleistocene settlement in a highly suitable lacustrine habitat in central Chile (34°S)

Labarca et al.

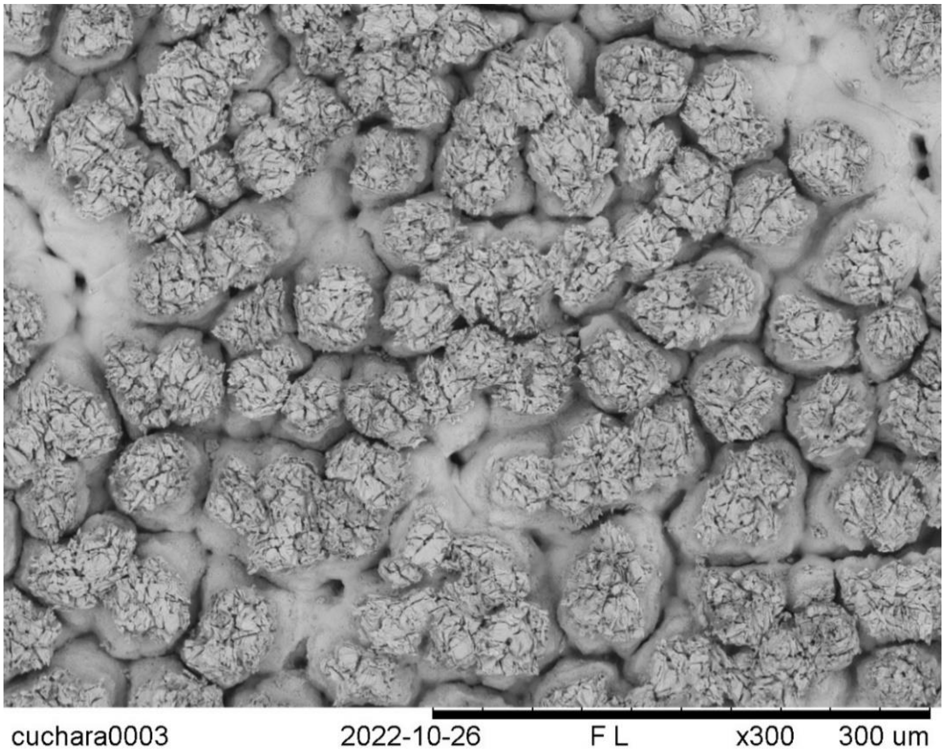

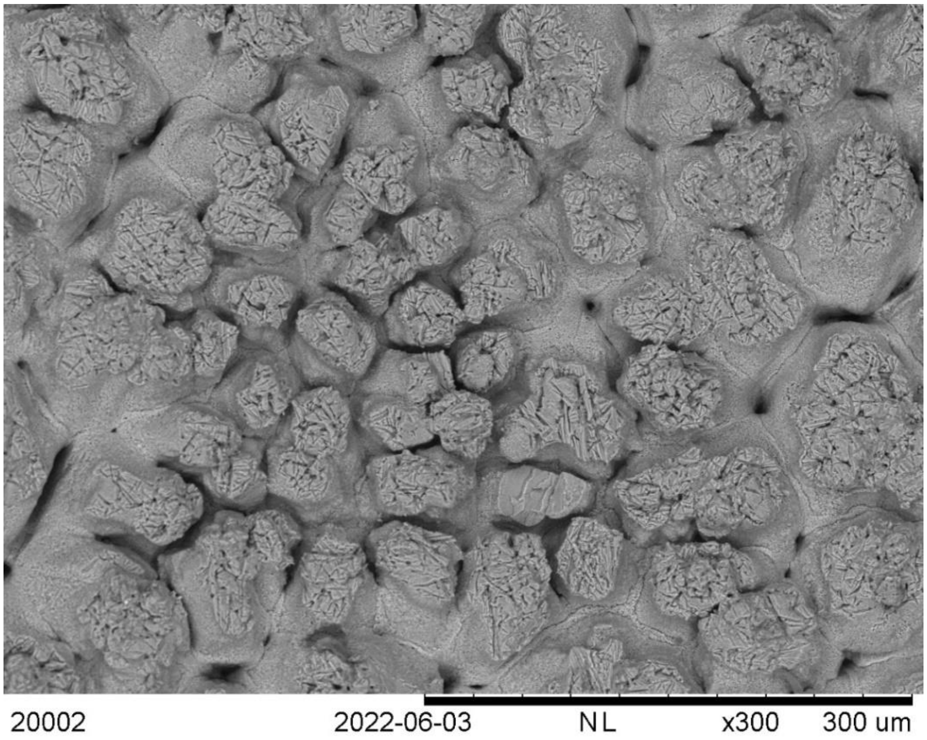

S3 Fig. Eggshells. Top: *Spatula clypeata* actual eggshell. Bottom: Archaeological eggshell fragment assigned to Anatidae undet. from archaeological unit D5, Level 18, Layer L4b.
