# Supplementary material for: Taguatagua 3: A new late Pleistocene settlement in a highly suitable lacustrine habitat in central Chile (34°S)
Source: PLoS One. 2024 May 22;19(5):e0302465. doi: 10.1371/journal.pone.0302465 (PMC11111044; doi:10.1371/journal.pone.0302465)
Supplement: S4 Fig — Source grouping follows Barbarena et al. (2019) and Sanhueza et al. (2022). Values obtained for artifact TT-F6-N18-01: Sr/Rb: 0.518; Rb/Zr: 0.473. Values obtained for Laguna El Maule samples: Sr/Rb: 0.526, 0.519, 0.537; Rb/Zr: 0,757; 0,804; 0,772, respectively. (PDF) [file pone.0302465.s004.pdf]

Taguatagua 3: a new late Pleistocene settlement in a highly suitable lacustrine habitat in central Chile (34°S)
Labarca et al.

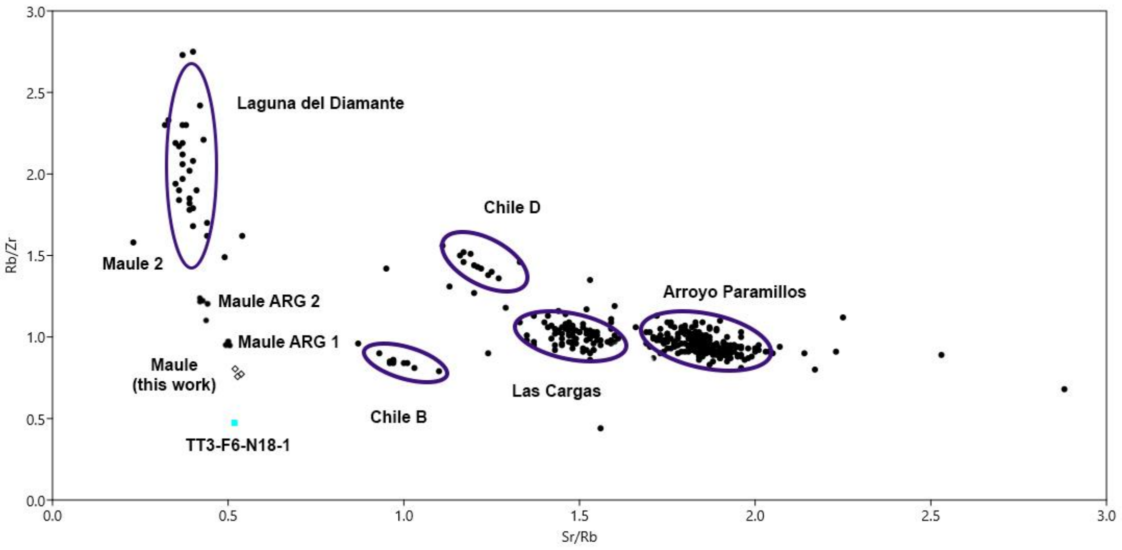

S4 Fig. Scatterplot of Sr/Rb versus Rb/Zr for obsidian artifacts from Mendoza and Central Chile. Source grouping follows Barbarena et al. (2019) and Sanhueza et al. (2022). Values obtained for artifact TT-F6-N18-01: Sr/Rb: 0.518; Rb/Zr: 0.473. Values obtained for Laguna El Maule samples: Sr/Rb: 0.526, 0.519, 0.537; Rb/Zr: 0,757; 0,804; 0,772, respectively.
