# Supplementary material for: Taguatagua 3: A new late Pleistocene settlement in a highly suitable lacustrine habitat in central Chile (34°S)
Source: PLoS One. 2024 May 22;19(5):e0302465. doi: 10.1371/journal.pone.0302465 (PMC11111044; doi:10.1371/journal.pone.0302465)
Supplement: S5 Fig — (PDF) [file pone.0302465.s005.pdf]

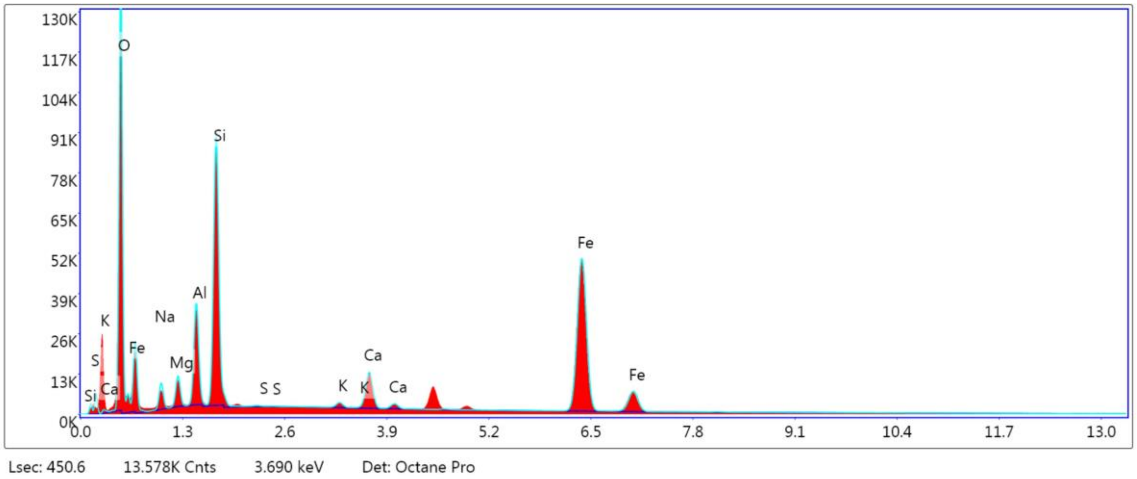

S5 Fig. SEM-EDX spectra of red particles inside a cavity of the lithic artefact.
