# Supplementary material for: Taguatagua 3: A new late Pleistocene settlement in a highly suitable lacustrine habitat in central Chile (34°S)
Source: PLoS One. 2024 May 22;19(5):e0302465. doi: 10.1371/journal.pone.0302465 (PMC11111044; doi:10.1371/journal.pone.0302465)
Supplement: S6 Fig — In red, the presence of iron oxide is more abundant inside the cavity than on the border. (PDF) [file pone.0302465.s006.pdf]

Taguatagua 3: a new late Pleistocene settlement in a highly suitable lacustrine habitat in central Chile (34°S)
Labarca et al.

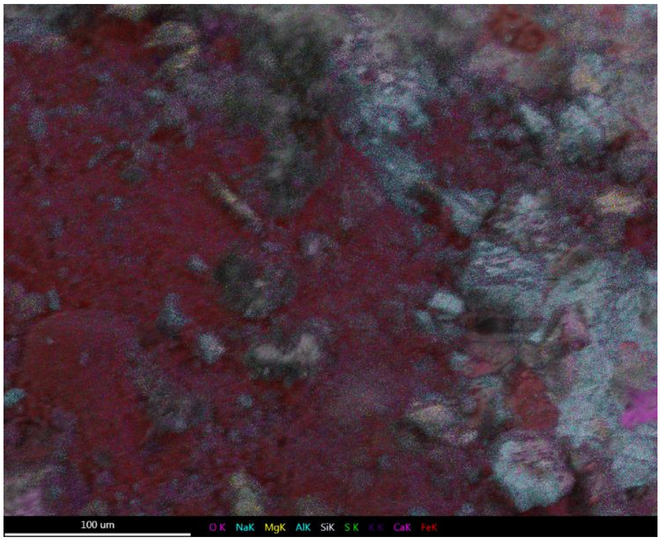

S6 Fig. Mapping image of inside and a border of a cavity on lithic artefact. In red, the presence of iron oxide is more abundant inside the cavity than on the border.
