# Supplementary material for: Taguatagua 3: A new late Pleistocene settlement in a highly suitable lacustrine habitat in central Chile (34°S)
Source: PLoS One. 2024 May 22;19(5):e0302465. doi: 10.1371/journal.pone.0302465 (PMC11111044; doi:10.1371/journal.pone.0302465)
Supplement: S8 Fig — a) Scirpus charred seeds. b) seed of cf. Elatinaceae c) Charred Portulacaceae fruit. d) Uncharted Portulacaceae seed and fruit. e) Cactaceae seed. f) Unidentifiable fruit. g) Fabaceae seed. h) Bromus sp. Inflorescence. i) Unidentified fruit. (PDF) [file pone.0302465.s008.pdf]

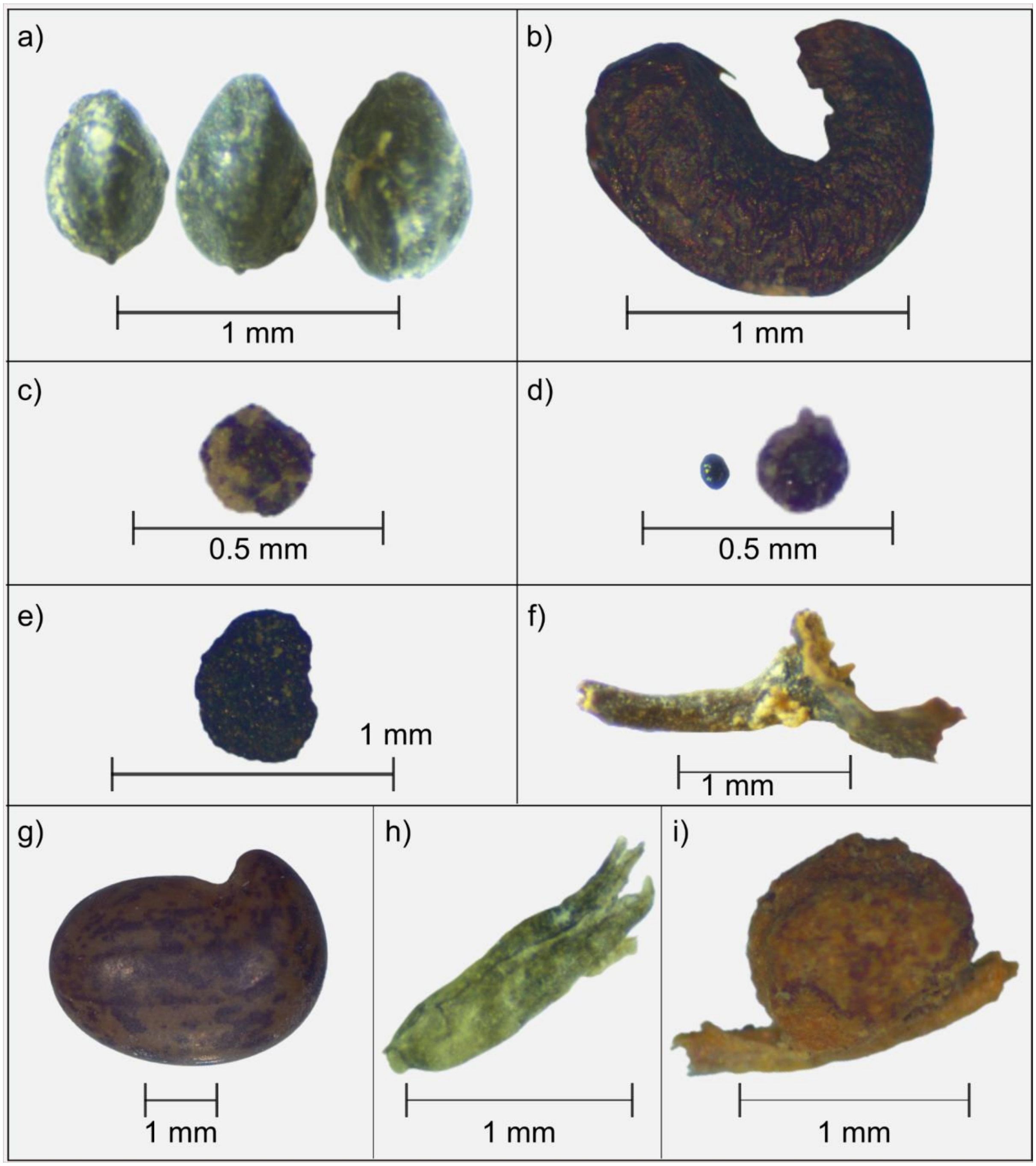

S8 Fig. Plant remains from TT-3 combustion feature. a) *Scirpus* charred seeds. b) seed of cf. Elatinaceae c) Charred Portulacaceae fruit. d) Uncharted Portulacaceae seed and fruit. e) Cactaceae seed. f) Unidentifiable fruit. g) Fabaceae seed. h) *Bromus* sp. Inflorescence. i) Unidentified fruit.
