# Supplementary material for: Taguatagua 3: A new late Pleistocene settlement in a highly suitable lacustrine habitat in central Chile (34°S)
Source: PLoS One. 2024 May 22;19(5):e0302465. doi: 10.1371/journal.pone.0302465 (PMC11111044; doi:10.1371/journal.pone.0302465)
Supplement: S1 File — (PDF) [file pone.0302465.s019.pdf]

Material and Methods

Archaeological excavations at TT-3 were carried out during four consecutive-year field seasons (2019-2022), resulting in 18m^2 being exposed. Initially, two adjacent test pits were excavated (archaeological Units 1 and 2: 2m^2), followed by an extensive intervention (16m^2, Fig 2 in the main text). Sediments were carefully extracted following the previously defined natural layers, combined with 10 cm thick artificial levels. The Late Pleistocene fertile deposit (facies L4b) was no more than 17 cm thick, but did not usually exceed 10-13 cm; nevertheless, to obtain a more controlled provenance of the artifactual and ecofactual materials, it was divided in two comparable artificial levels: upper and lower. Since the whole sedimentary pile has a natural orientation tilt towards the south and west (from the lake's center), this facies appears at different depths around the undisturbed areas: around 1.58 m in the north area (excavation unit F6) and 1.75 in the south (excavation unit B6). In some excavation units in the west area (excavation units B4 to G4), especially those affected by landslides (see the Chronostratigraphic Framework and S2 File), a small portion of the L4b facies was extracted from a third level (either levels 19 or 20), but the archaeological material was included in the lower level.

We conducted a tridimensional record of all bone fragments, lithic materials, and features of >5 cm. The latter allowed for the piece-plotting of 182 individual specimens in the late Pleistocene facies. All features were recovered for flotation and sediment analysis. The sediments obtained were wet-sieved, using 2 mm screen meshes. Considering only the Late Pleistocene facies, the total number of specimens recovered account for 20,626 units (20,353 bone specimens, 241 malacological fragments, 17 eggshell fragments, and 15 lithics).

Three new stratigraphic columns were constructed at TT-3: TT19-A3-1E (1.80 m thickness), located in excavation unit 1; TT20-B3-1E (1.68 m thickness) in excavation unit D5, and TT22-C3-1E (1.78 m thickness) in excavation unit F6 (Fig 3 in the main text). Sedimentary facies were defined using macroscopic visual characteristics that considered color, texture, and sedimentary structures, as well as elemental geochemistry data, their response to Hydrogen Chloride (HCl), and their fossil content. To standardize the macroscopic description of the facies, we adopted the lake sediments classification scheme of Schurrenberger et al. (2003). Facies were grouped into units according to Miall (2000), which combines biogeochemical parameters with distinctive lithologic features of

sedimentary facies (see Frugone-Álvarez et al., 2023). Facies and units were originally described in TT19-3A-1E and then correlated with stratigraphic columns TT20-B3-1E and TT22-C3-1E from later excavations (2020-2022).

The chronology of the site was reconstructed from ten AMS-radiocarbon dates on charcoal and bulk sediments taken from the TT19-A3-1E column, plus six charcoal samples taken from the archaeological excavation. All samples were processed at the DirectAMS laboratories in Bothell, WA, USA. Results are presented in units of percent modern carbon (pMC) and the uncalibrated radiocarbon age before present (relative to 1950 CE). Radiocarbon ages were calibrated using the Southern Hemisphere calibration curve (Hogg et al., 2020). We used the "rbacon" package v3.1.1 (Blaauw and Christen, 2013) in R v4.3.1 (R Core Team, 2021) to establish an age-depth model and the deposition rates along the sequence.

Faunal remains from all the excavation units were divided into two categories without formal taxonomical value: small vertebrates (<15 kg) and megafauna (>15 kg), considering their morphology, size, and thickness. Small vertebrates included birds, amphibians, rodents, and fish, while megafauna included proboscideans, equids, and cervids. Small vertebrates are currently under taxonomical description, so only a Class/Order identification level is presented here. On the other hand, megafaunal remains were identified to the most inclusive level. Fossils were anatomically and taxonomically identified using reference texts (i.e, Barone, 1976; Baumel and Witmer, 1993; Reed, 1974; Reig, 1977) and current reference collections as well as fossil ones (Museo de Historia Natural de Santiago de Chile and Douglas Jackson personal collection). Several quantification units were used, including NSP (number of specimens), NISP (number of identified specimens), MNE (minimal number of elements), MAU (minimal anatomical units), %MAU and %RA (relative abundance) (Andrews, 1990; Binford, 1978; Grayson, 1984; Lyman, 2008). For rodents, birds, and anurans, we evaluated their anatomical proportions based on indexes from Bisbal-Chiniesta et al. (2020), Ericson (1987), Bochenski and Nekrasov (2001), Bochenski (2005), Andrews (1990), and Fernández-Jalvo and Andrews (1992). Regarding taphonomy, all identified megafaunal remains were considered, while only a subsample of unidentified/cranial megafaunal specimens and small vertebrates from units C5, B4, and B5 were studied. In both cases, remains were inspected under a low-magnification stereomicroscope (35x), to search for surface modifications. These included cutmarks, fire marks, percussion marks, digestive traces (mainly pitting), tooth marks, root

etching, weathering, polishing, and manganese coating (Andrews, 1990; Behrensmeyer, 1978; Binford, 1980; Cáceres et al., 2002; Fernández-Jalvo and Avery, 2015; Fernández et al., 2017; Mengoni-Goñalons, 1999; Lyman, 1994, among others). The intensity and location of each modification were also recorded (Lizama-Catalán and Labarca, 2023).

A small sample of archaeological eggshells (NISP = 3) from Unit D5 was exploratorily analyzed. The samples were cleaned using an ultrasonic tank filled with distilled water and then macro and microscopically identified using a modern data set of local taxa. Using a Scanning Electron Microscope (SEM) (Hitachi TM3000) and different high-power magnification (100x, 200x, 300x, and 800x), we described and counted the number of pores and their distribution, as well as the number of mammillae, their definition, shape, space, and distribution. The thickness of each fragment was also recorded (Hicks et al., 2023; Sidell, 1993; Taivalkoski et al., 2022).

The lithic assemblages from all excavation units were morphologically and techno-functionally studied following Bate (1971), Jackson (2002), Andrefsky (2000), and Odell (2003). Raw material was macroscopically classified using color, grain size, and inclusions as a general reference. Regarding their provenance, due to the very limited knowledge about the regional distribution of lithic resources (Méndez and Jackson, 2015), rocks were tentatively divided into local, possibly exotic, and exotic groups. We followed the Meltzer (1989) classification, which suggested more than 40 km for an exotic raw material, although this limit may be open to discussion for different cases. For obsidian provenance specifically, an elemental analysis was performed using a portable XRF (X- Ray Fluorescence) Bruker Tracer 5G series. The Rh anode transmission X-ray tube was operated at 40 KeV, with a current flow of 75 uA for 111 seconds of exposure to obtain optimal resolution. The instrument spot was 0.3 mm. The program ARTAX 7 was used to analyze the spectral information. We also analyzed the artifact TT3-F6-N18-01 and two reference samples from Laguna del Maule source (Velozo 1988). Following the literature (i.e., Glascock et al., 1998; Sanhueza et al., 2021), the proportion of strontium-rubidium and rubidium zirconium was used to analyze the results. Empirical data from Barberena et al. (2019) and Sanhueza et al. (2021) was employed for comparison.

In technological terms, metric variables were recorded in millimeters. We also recorded fragmentation, the original blank inferred of each specimen, and the length and morphology of flaking. For flakes, we identified the morphology of the striking platform, the cortex percentage on

the dorsal face, and its stage on the reduction sequence (Andrefsky, 2000; Odell, 2003). Finally, lithic artifact categories followed Bate (1971) and Jackson's (2002) classification schemes. All the lithic specimens were observed under a stereomicroscope (up to 35x) looking for use-wear and/or post depositional modifications. The presence/absence and morphological attributes of flaking scars, polishing, rounding, and striae were also recorded (Mansur-Franchomme, 1987, 1999). A subsample of two specimens was preliminarily inspected under high magnification (100x and 200x) using a Zeiss Axioscope microscope. We also recorded the absence/presence and description of micro-polishing, linear features, rounding of edges, and attached residues for this assemblage (Acosta et al., 2013; Keeley, 1980; Mansur-Franchomme, 1999; Rots et al., 2015).

With respect to pigment analysis, after microscopic observations, an SEM examination coupled with an Energy Dispersive X-ray spectroscopy (SEM-EDX) analysis were carried out, while an FEI Quanta FEG 250 microscope was used to capture images and elemental spectra. Raman's spectroscopy was employed to determine the pigments' molecular characterization. Spectra samples were recorded on a WITec Alpha 300 RA confocal microscope, with a laser excitation wavelength of 532 and 785 nm. The optical resolution of the machine was ~200 nm in its lateral axes. The instrument was calibrated using the 520 cm^{-1} line of a Si wafer and a 50X objective, with the same working conditions. The system's resolution was 0.2 cm^{-1}, with 128 scans; spectra were recorded in the 200–800 cm^{-1} region at a laser wavelength of 785 nm and by using a 600 g/mm^{-1} grating to observe the Raman spectra. The spectral scanning conditions were chosen to avoid sample degradation and photodecomposition. Data was collected and plotted using WIRE 3.4 and Origin Lab Pro 2016 software.

Regarding archaeobotanical remains from the combustion feature, a total of 5 liters of bulk soil sample was processed through an assisted flotation machine system (Greig, 1989; Watson, 1979). The observation and separation of the lighter fraction was performed using a trinocular stereomicroscope, with magnifications of up to 35x. The heavy fraction was first screened with the naked eye and subsequently observed under stereomicroscope.

The materials recovered from the sediment were divided into major categories for analysis: bone remains, crustaceans, insects, and plants (the latter subdivided into charcoal, fruits, inflorescence, and seeds). The plant remains were separated into charred and non-charred. Fruits, inflorescence, and seeds were all classified into identified, unidentified, and unidentifiable. Plant remains were

identified using botanical reference collections at the Archaeology Laboratory and the Paleoecology and Paleoenvironments Laboratory of Pontificia Universidad Católica de Chile.

Charcoal fragments from the combustion feature were manually separated for analysis. Only well-preserved specimens (with well-defined cellular structures) greater than 2 mm were studied considering their three main anatomical views: transversal, longitudinal, and tangential longitudinal planes using an optical microscope. Taxonomic identification was performed using reference collections (Archaeobotany and Environmental History Laboratory of the Universidad Austral de Chile) and specialized bibliography (Rancusi et al., 1987; Schweingruber, 1990; Solari, 1993; Wagemann, 1949). Two samples identified at the family level were observed using SEM (Hitachi 53400N).

References

1. Andrefsky W Jr. Lithics: Macroscopic approaches to analysis. United Kingdom: Cambridge University Press. 2000. doi: 10.1017/CBO9780511810244.

2. Andrews P. Owls, caves and fossils: predation, preservation and accumulation of small mammal bones in caves, with an analysis of the Pleistocene cave faunas from Westbury-sub-Mendip, Somerset, UK. University of Chicago Press. 1990.

3. Barberena R, Fernández MV, Rughini AA, Borrazzo K, Garve, Lucero G, et al. Deconstructing a complex obsidian "source-scape": A geoarchaeological and geochemical approach in northwestern Patagonia. Geoarchaeology. 2019;34(1):30-41. doi: 10.1002/gea.21701.

4. Barone R. Anatomie comparée des animaux domestiques: Ostéologie. Vigot, Paris, France. 1976.

5. Bate LF. Material lítico: metodología de clasificación. Noticiario Mensual Museo Historia Natural (Chile). 1971;181:1-23.

6. Baumel JJ, Witmer LM. Osteologia. In: Baumel JJ, editor. Handbook of avianan atomy: Nomina Anatómica Avium. Cambridge Nutall Ornithological Club. 1993. p. 45–132.

7. Behrensmeyer AK. Taphonomic and ecologic information from bone weathering. Paleobiology. 1978;4(2):150-62. doi: 10.1017/S0094837300005820.

8. Binford LR. Nunamiut ethnoarchaeology. New York, Academic Press. 1978.

9. Binford LR. Willow smoke and dogs' tails: hunter-gatherer settlement systems and archaeological site formation. American Antiquity. 1980;45(1):4-20. doi: 10.2307/279653.

10. Blaauw M, Christen JA. Bacon Manual v2. 3.3. Queens University: Belfast, UK. 2013.

11. Bocheński ZM. Owls, diurnal raptors and humans: signatures on avian bones. In: O'Connor T, editor. Biosphere to lithosphere. New studies in vertebrate taphonomy. 2005. p. 31-45.

12. Bochenski ZM, Nekrasow AE. The taphonomy of sub-Atlantic bird remains from Bazhukovo III, Ural Mountains, Russia. Acta zoologica cracoviensia. 2001;44(2):93-106.

13. Cáceres I, Bravo P, Esteban M, Expósito I, Saladié P. Fresh and heated bones breakage. An experimental approach. In: de Renzi M, Pardo Alonso M, Belinchón M, Peñálver E, Montoya P, Márquez-Aliaga A, editors. Current topics on taphonomy and fossilization. 2002. p. 471–9.

14. Ericson PG. Interpretations of archaeological bird remains: a taphonomic approach. Journal of archaeological science. 1987;14(1):65-75. doi: 10.1016/S0305-4403(87)80006-7.

15. Fernández-Jalvo Y, Andrews P. Small mammal taphonomy of Gran Dolina, Atapuerca (Burgos), Spain. Journal of Archaeological Science. 1992;19(4):407-28. doi: /10.1016/0305-4403(92)90058-B.

16. Fernández-Jalvo Y, Avery DM. Pleistocene micromammals and their predators at Wonderwerk Cave, South Africa. The African Archaeological Review. 2015:751-91. doi: 10.1007/s10437-015-9206-7.

17. Fernández FJ, Montalvo CI, Fernández-Jalvo Y, Andrews P, López JM. A re-evaluation of the taphonomic methodology for the study of small mammal fossil assemblages of South America. Quaternary Science Reviews. 2017;155:37-49. doi: 10.1016/j.quascirev.2016.11.005.

18. Glascock MD, Braswell GE, Cobean RH. A systematic approach to obsidian source characterization. Archaeological obsidian studies: method and theory. 1998: 15-65. doi: 10.1007/978-1-4757-9276-8_2.

19. Grayson DK. Quantitative Zooarchaeology: Topics in the Analysis of Archaeological Faunas. Academic Press, New York. 1984. doi: 10.1016/C2009-0-21855-1.

20. Greig J. Archaeobotany. Handbook for Archaeologist No. 4. Strasbourg: European Science Foundation. 1989.

21. Hicks M, Anamthawat-Jónsson K, Einarsson Á. The identification of bird eggshell by scanning electron microscopy. Journal of Archaeological Science. 2023;151:105712. doi: 10.1016/j.jas.2022.105712.

22. Hogg AG, Heaton TJ, Hua Q, Palmer JG, Turney CS, Southon J, et al. SHCal20 Southern Hemisphere calibration, 0–55,000 years cal BP. Radiocarbon. 2020;62(4):759-78. doi: 10.1017/RDC.2020.59.

23. Jackson D. Los instrumentos líticos de los primeros cazadores de Tierra del Fuego. Santiago, Centro de Investigación Diego Barros Arana. 2002.

24. Keeley LH. Experimental Determination of Stone Tool Uses: A Microwear Analysis. University of Chicago Press, Chicago. 1980.

25. Lyman RL. Quantitative paleozoology. Cambridge, Cambridge University Press. 2008. doi: 10.1017/CBO9780511813863.

26. Mansur-Franchomme ME. El análisis funcional de artefactos líticos. Buenos aires, Cuadernos Serie Técnica 01, Instituto Nacional de Antropología. 1987.

27. Mansur ME. Análisis funcional de instrumental lítico: problemas de formación y deformación de rastros de uso. Proceedings of the XII Congreso Nacional de Arqueología Argentina; 1997 Sep 22-26; La Plata, Argentina. 1999. p. 355-66.

28. Meltzer DJ. Was stone exchanged among eastern north American paleoindians? In: Ellis CJ, editor. Eastern Paleoindian Lithic Resource Use. Routledge. 1989. p. 11-39. doi: 10.4324/9780429049743.

29. Mengoni Goñalons GL. Cazadores de guanacos de la estepa patagónica. Buenos Aires, Sociedad Argentina de Antropología. 1999.

30. Odell G. Lithic Analysis. Manuals in Archaeological Method, Theory, and Technique. Springer. 2003. doi: 10.1007/978-1-4419-9009-9.

31. R Core Team. R: a language and environment for statistical computing. R Foundation for Statistical Computing, Vienna. 2021.

32. Rancusi MH, Nishida M, Nishida H. Xylotomy of important Chilean woods. Contributions to the Botany in the Andes II. 1987;2:68-154.

33. Reed C. Archaeology: Osteology for the Archaeologist. Number 3, The American Mastodon and the Woolly Mammoth; Number 4, North American Birds: Skulls and Mandibles; Number

5, North American Birds: Postcranial Skeletons. In: Olsen S, editor. American Anthropologist. Peabody Museum Press. 1974;76. p. 945-6. doi: 10.1525/aa.1974.76.4.02a00860.

34. Reig OA. A proposed unified nomenclature for the enamelled components of the molar teeth of the Cricetidae (Rodentia). Journal of Zoology. 1977;181(2):227-41. doi: 10.1111/j.1469-7998.1977.tb03238.x.

35. Rots V, Hardy BL, Serangeli J, Conard NJ. Residue and microwear analyses of the stone artifacts from Schöningen. Journal of Human Evolution. 2015;89:298-308. doi: 10.1016/j.jhevol.2015.07.005.

36. Schnurrenberger D, Russell J, Kelts K. Classification of lacustrine sediments based on sedimentary components. Journal of Paleolimnology. 2003;29:141-54. doi: 10.1023/A:1023270324800.

37. Schweingruber FH. Anatomy of European woods. An Atlas for the Identification of European Trees, Shrubs and Dwarf Shrubs. Swiss Federal Institute for Forest, Snow and Landscape Research series on scientific topics. 1990.

38. Sidell EJ. A methodology for the identification of archaeological eggshells. UPenn Museum of Archaeology. 1993;10.

39. Solari ME. L'Homme et le bois en Patagonie et Terre de Feu au cours des six derniers millénaires: recherches anthracologiques au Chili et en Argentine [dissertation]. Université Montpellier-II, France. 1993.

40. Taivalkoski A, Holt E, MacKinnon M. Bird eggs in the diet of ancient Pompeii: An SEM analysis of archaeological avian eggshell. Journal of Archaeological Science: Reports. 2022;41:103258. doi: 10.1016/j.jasrep.2021.103258.

41. Wagemann G. Maderas chilenas, contribución a su anatomía e identificación. Lilloa. 1949;16:263-376.

42. Watson PJ. In pursuit of prehistoric subsistence: a comparative account of some contemporary flotation techniques. Midcontinental Journal of Archaeology. 1976;1:77–100.
