# Supplementary material for: Taguatagua 3: A new late Pleistocene settlement in a highly suitable lacustrine habitat in central Chile (34°S)
Source: PLoS One. 2024 May 22;19(5):e0302465. doi: 10.1371/journal.pone.0302465 (PMC11111044; doi:10.1371/journal.pone.0302465)
Supplement: S2 File — (PDF) [file pone.0302465.s020.pdf]

Taguatagua 3: a new late Pleistocene settlement in a highly suitable lacustrine habitat in central Chile (34°S)
Labarca et al.

Supplementary File 2

Description of post-depositional alterations on the west area of the TT-3 site

Only on the western part of the excavation (units B4 to G4, Figure 2 and Figure 10 on main text), we recorded a post-depositional process that slightly altered the normal disposition of the facies on the sequence. In the north and south profiles (transversal to the drainage channel axis), we observed the development of parallel cracks with a westward dip, which exhibit a stepped displacement of about 10 cm of the stratification surfaces, most clearly evidenced in the contacts between the Units 4 and 5 (Figure 1). The area comprised by this alteration is about 14% of the overall surface excavated (ca. 2,5 m^2). This feature was identified as landslides originated by erosion of the channel that drained the lake since its construction during the XIX century. Landslides normally have a down slope orientation, but eventually inverse movements are expected along the planes of weakness, forming pillars that locally displace the layers only a few centimeters upwards. Consequently, in the west portion of these units, the artifactual and ecofactual materials may appear at greater depths in relation to adjoining units. However, this type of movement does not disturb the horizontal distribution of the materials, but only slides the sequence, between cracks, a few centimeters downwards, which means that materials are still associated in their original facies and within their sediments.

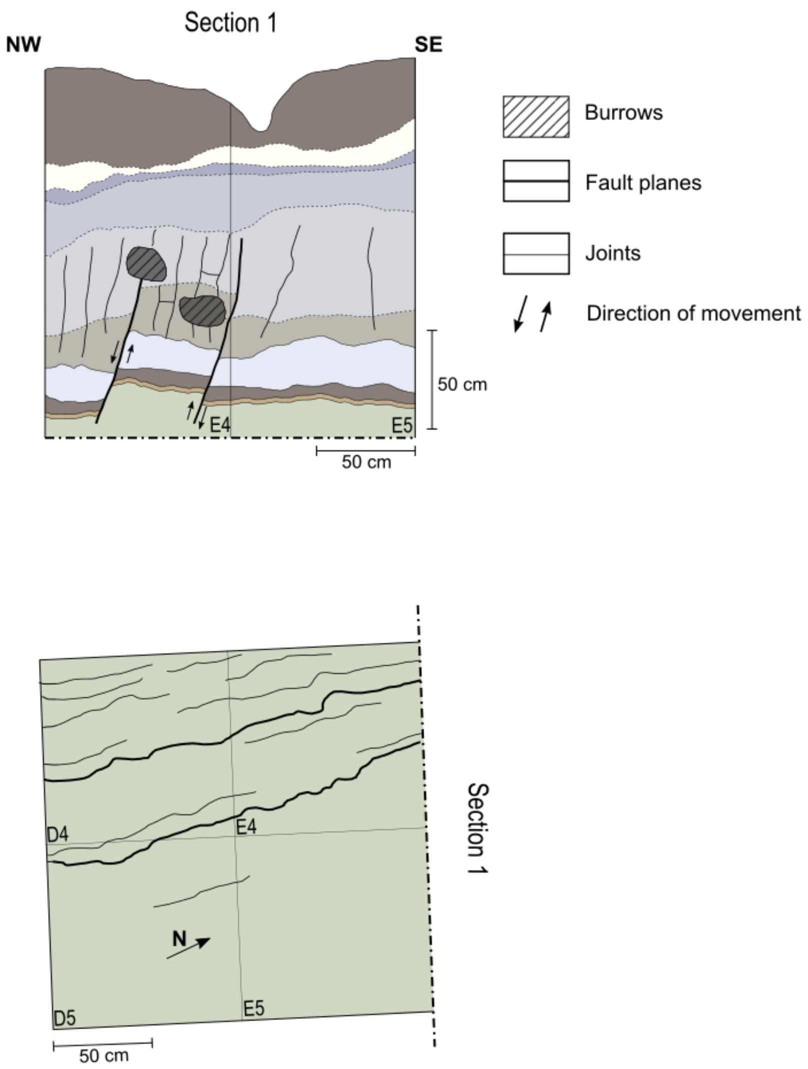

Figure 1. A. Detail of the north profile of Units E4 and E5 (simplified), showing the variations in the stratigraphy due the landslides. B. Schematic plan of the Units E4, E5, D4 and D5, showing the extension of the landslides.

Taguatagua 3: a new late Pleistocene settlement in a highly suitable lacustrine habitat in central Chile (34°S)
Labarca et al.

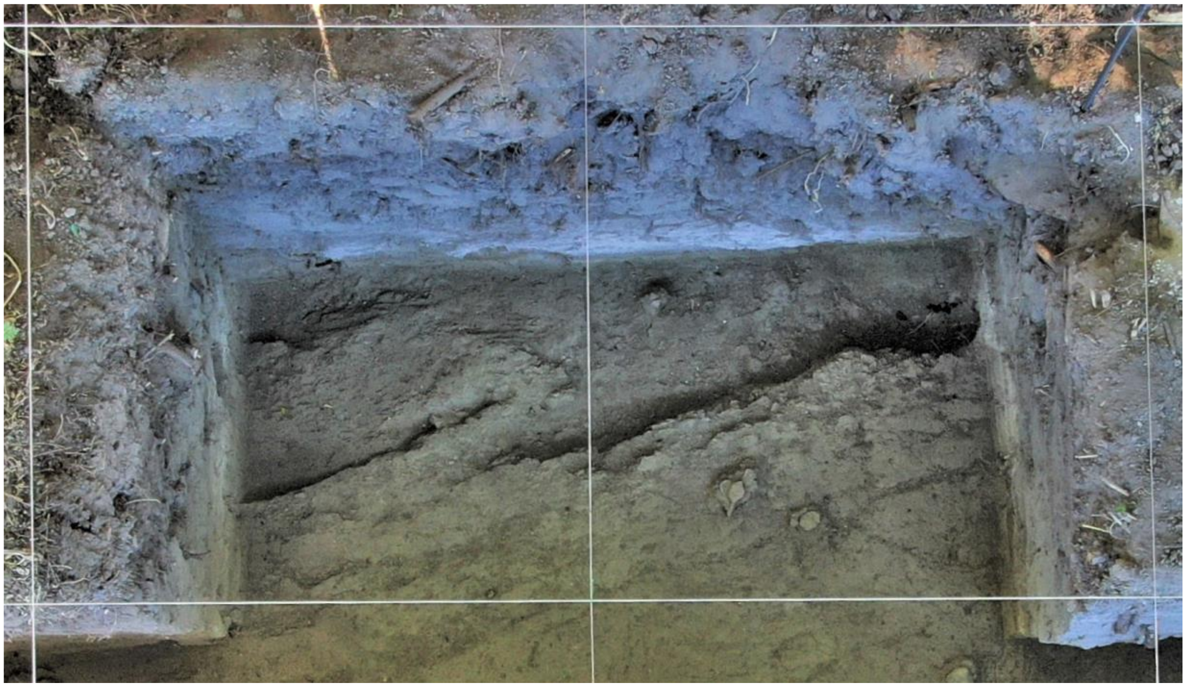

Figure 2. Photography of Units D4, D5, E4 and E5 with the extension of the landslide.
