# Supplementary material for: Taguatagua 3: A new late Pleistocene settlement in a highly suitable lacustrine habitat in central Chile (34°S)
Source: PLoS One. 2024 May 22;19(5):e0302465. doi: 10.1371/journal.pone.0302465 (PMC11111044; doi:10.1371/journal.pone.0302465)
Supplement: S3 File — (PDF) [file pone.0302465.s021.pdf]

Supplementary File 3

Origin of the small vertebrate assemblage at TT-3

Since small vertebrates were recovered indirectly from both upper and lower levels of facies L4b, it was assumed that in the former, small vertebrates were mainly deposited naturally, and in the latter, human agency was responsible for at least a portion of the assemblage. The analysis of the taxa diversity, frequencies of skeletal parts and taphonomic modifications in the lower level, and its comparison with the upper one, seems to corroborate this assumption.

Birds are particularly important in the lower level, and at TT-1 the most intensive human exploitation was detected mainly in this group (Lizama-Catalán and Labarca, 2023). At both sites, big taxa such as Anatidae (>300 g) were more predominant. In central Chile, bird consumption by raptors has been recorded for falconiforms, but they usually prey upon small taxa (<150 g), such as Passeriforms (i.e., *Elaenia albiceps* or *Turdus falcklandii*) (Figueroa et al., 2004; Figueroa and Corales, 2004). Rodents, and particularly Octodontids, are also well represented in the lower-level subsample and nearly absent in the upper one, as are the most common rodent group identified in TT-1 (Lizama-Catalán and Labarca, 2023). Octodontids had been exploited by hunter-gatherers in the Andean range of central Chile during the Holocene (Simonetti and Cornejo, 1991) as well as during early historic times (de Ovalle, 1974). Rodents are also a common prey of several diurnal and nocturnal raptors and carnivores in central Chile, but usually they combine Caviomorphs (mainly families Abrocomidae and Octodontidae) with small cricetids such as *Pyllothis darwini* or *Oryzomys longicaudatus* (i.e., Herrera and Jaksic, 1980; Jaksic et al., 1980a; Muñoz-Pedreros et al., 2017; Jimenez and Jaksic, 1989; Schlatter et al., 1980). Some falconiforms and foxes prey mainly on medium-sized rodents, such as octodontids (i.e., Schlatter et al., 1980), but if the rodent assemblage in the lower level was mainly the result of the deposition of scats and/or diurnal raptor pellets, gastric acids should reflect this taphonomic scenario, which is not the case (see below).

On the other hand, anurans are best represented in the upper level, and this sample is dominated by immature individuals, in contrast with the lower level. A high subadult mortality has been suggested for *Calyptocephalella*, due it's particularly extensive premetamorphic age, coupled with the large quantity of eggs that females lay in each spawning (around 16,000) (Vélez, 2019; Vélez and Acuña, 2012). An age profile dominated by adults would be expected in an anthropogenic assemblage (Bailon, 1997; Bisbal-Chiniesta et al., 2020), which coincides with the record of the lower level. Finally, fish are well represented in both levels, but they are more common in the upper level.

Frequencies of skeletal parts are not fully conclusive. In rodents, cranial remains as well as long bones are more represented than vertebrae and ribs, which can be explained by the fragility and/or size of the latter group of bones. Interestingly, there are more postcranial than cranial bones, a feature that has been observed in South American nocturnal raptor pellets. The relatively similar frequencies of cranial and proximal limbs were also observed in this group (Montalvo et al., 2016;

Gómez, 2005, 2007; López 2020; Montalvo and Tejerina, 2009). A high proportion of postcranial to cranial remains have been observed in the pellets of diurnal raptors *Geranoaetus melanoleucus* as well, but the opposite pattern was suggested for other diurnal raptors, such as *Elanus leucurus* and *Geranoaetus polyosoma* (Montalvo et al., 2016; López et al., 2017). Information from mammalian carnivore scats (*Leopardus geoffroyi*, *Lontra longicaudis,* and *Lycalopex griseus*) suggested a predominance of cranial elements but there were more proximal than distal remains (Montalvo et al., 2012, 2015; Gómez and Kaufmann, 2007). The pattern observed in the TT-3 rodent sample is not concordant with the information about human consumption. In central Chile, Simonetti and Cornejo (1991), and in northern Patagonia, Andrade and Fernández (2017), have pointed out that rodent anthropogenic assemblages are characterized by a predominance of cranial remains, and, to a lesser extent, distal-limb bones. Simonetti and Cornejo (1991) indicate that this pattern is related to processing techniques, as cranial and mandibular bones are removed (and discarded) before or after being cooked.

Frequencies of birds' skeletal remains indicate a low proportion of cranial remains and a well-represented vertebral column. Leg bones are more represented than wing bones, and there are more limb than core bones. This pattern is not coincident with taphonomic literature. For example, a high frequency of wing bones is linked to non-human predation, especially those of diurnal birds (Bochenski and Tomek, 1997; Bochenski, 2005; Bochenski et al., 2009, 2021; Laroulandie, 2002; Lloveras et al., 2017). Leg bones are more common than wing bones in samples generated by mammalian carnivores (Rodríguez-Hidalgo et al., 2016) but high frequencies of limb bones have been related to human exploitation as well (Ericson, 1987). At TT-1, anatomical studies indicate that meat-rich bones such as coracoid, scapula, furcula, humerus, and sternum are more represented than limb elements, which is not the case here (see also Bochenski et al., 2018).

Regarding anurans, cranial bones, especially frontoparientals and exooccipitals, are the most recorded elements. Pelvises are relatively well represented; limb bones are not. Forelimb bones are more represented than hindlimb bones, and more distal limb bones have been recorded than proximal ones. The opposite pattern has been suggested for pellets from *Tyto furcata* in which cranial remains are scarce and hindlimbs are more predominant than forelimbs (Mignino et al., 2021). In *Athene cunicularia*, another strigiform, cranial and postcranial bones are relatively equally represented (Mignino et al., 2021). No detailed information is available for other predators such as falconids or mammals. Nor does the pattern observed in TT-3 fit with anthropogenic accumulations, given that a high presence of the hindlimbs and pelvic girdle would be expected due to their higher meat content (i.e., Bailon, 1997; Chiquet, 2005; Kysely, 2008). Remarkably, the frequencies observed at TT-3 are very similar to those reported for TT-1 (Lizama-Catalán and Labarca, 2023).

Regarding taphonomic modifications, the incidence of gastric corrosion in the lower level is markedly low (2.3% of the sample analyzed). This value is considerably lower than that of the TT-1 sample (16.8%) and in general lower than in any actualistic references, considering birds, anurans and rodents (Andrews, 1990; Montalvo et al., 2016; Bochenski and Tomek, 1997; Bochenski et al., 2009, 2021; Pinto Llona and Andrews, 1999, among others). This suggests that birds and carnivores

were not the main depositional agent at TT-3. Nevertheless, several differences were observed among the four main taxonomic categories. No pitting marks were observed in fish, and, in contrast, anurans are the most affected group (NISP = 7%; MNI = 3; 33.3% of the total MNI). The latter also exhibits the highest intensity of gastric corrosion (severe and extreme), which could be linked, either to diurnal raptors, and/or carnivores. In this regard, *Calyptocephallela* remains have been documented in *Lontra provocax* spraints (Medina-Vogel and González-Lagos, 2008), and it is possible to speculate that *Lycalopex culpaeus* could hunt more vulnerable subadults as well, considering their ability to prey upon juvenile lagomorphs (Jaksic et al., 1980b). Nevertheless, it must be highlighted that the proportion of immature toads with acid marks in this level is lower than in the upper one. On the other hand, gastric corrosion in birds and rodents is even lower, and the low intensity recorded is coherent with diurnal raptors' pellets.

Cut marks are a clear indicator of human agency, but they are extremely infrequent in the sample analyzed. These results contrast with the values recorded at TT-1, where cutmark frequencies and their locations helped to reconstruct how the birds were processed (Lizama-Catalán and Labarca, 2023). The locations of the traces recorded in TT-3 birds' scapula are similar to those recorded at TT-1 and could be explained by the disarticulation of a rich-meat segment. The presence of cutmarks in the anuran diaphysis of a tarsal cannot be associated with a disarticulation or fileting activity. We hypothesize that its location is more coherent with skinning activities. No cut marks were observed in rodents, but this absence was also observed at TT-1 (Lizama-Catalán and Labarca, 2023). Fire marks on small vertebrates were certainly generated by humans. Natural fires produce a homogeneous chromatic profile, usually dominated by intensive stages of fire exposure (whitish or grayish) (i.e., Álvarez et al., 2017; Weihmüller et al., 2022). At TT-3, fire action on small vertebrates affected 12.5% of the sample from the lower level, and figures are even lower in the upper level (7.8%). Also, there are differences in the frequencies of burned bones among the general taxonomic categories, and, internally, each category showed differences in the coloring of the bones as well, indicating that the specimens were exposed to different temperatures and/or time periods. These features clearly rule out a natural burning scenario and even anthropogenic fireplaces situated over previously deposited fauna (Bennet, 1999). In this regard, rodents and birds are the most burned group, in contrast to anurans and fish. In general terms blackish coloration is the most common stage, which indicates moderate heat exposure events (ca. 500°C) (Shipman et al., 1984; Stiner et al., 1995; Cáceres et al., 2002). Megafaunal remains were comparatively more burned (78.2%), but the bones had been exposed for a reduced time and/or to a gentle heat source.

In sum, taphonomic and taxonomic information suggest that at least a portion of the small vertebrate assemblage in the lower level of facies L4b was anthropically introduced. If the incidence of fire and gastric corrosion is considered as a proxy for human exploitation and non-human predation, respectively, rodents and birds would have been more exploited than anurans and fish. However, cut-marked anuran bones, as well as the age profile, comprising mainly adult individuals, suggest a minor anthropic exploitation as well, but mixed with natural depositions (i.e., carnivore scats and/or falconid pellets). Regarding fish, the absence of cutmarks and gastric corrosion, along with the low incidence of thermal alteration, suggest that this group entered the deposit mainly via

natural deaths. In this respect, the fish sample in the lower level exhibits the highest proportion of MnO_2 and abrasion marks, and coincidently, the lowest proportion of root etching. This taphonomic scenario would be consistent with a water-saturated context that affected the bones when the water table was high and is therefore inconsistent with human settlement.

References

1. Álvarez MC, Massigoge A, Scheifler NA, Gonzalez ME, Kaufmann CA, Gutierrez MA, Rafuse DJ (2017) Taphonomic effects of a grassland fire on a modern faunal sample and its implications for the archaeological record. Journal of Taphonomy 15(1–3), 77–90.

2. Andrade A, Fernández P (2017) Rodent consumption by hunter-gatherers in north Patagonian Andean forests (Argentina): insights from the small vertebrate taphonomic analysis of two late Holocene archaeological sites. Journal of Archaeological Science. Reports 11, 390-399.

3. Andrews PJ (1990) Owls, Caves and Fossils: Predation, Preservation and Accumulation of Small Mammal Bones in Caves, with an Analysis of the Pleistocene Cave Faunas from Westbury-sub-Mendip. University of Chicago Press, Somerset, UK

4. Bailon S (1997) La grenouille rousse (*Rana temporaria*). Une source de nourriture pour les habitants de Chalain 3. In: Petrequin P (Ed.), Les sites littoraux neolithiques de Clairvaux-les-Lacs et de Chalain (Jura). III: Chalain 3, 3200-2900 av. J.-C, vol. 2. Editions de la Maison des sciences de l ’homme, Paris, pp. 711-716.

5. Bennett JL (1999) Thermal alteration of buried bone. Journal Archaeological Science 26 (1), 1-8.

6. Bisbal-Chiniesta JP, Banuls-Cardona S, Fernández-García M, Cáceres I, Blain HA, Verges JP (2020) Elucidating anuran accumulations: massive taphocenosis of tree frog Hyla from the Chalcolithic of El Mirador cave (Sierra de Atapuerca, Spain). Journal of Archaeological Science. Reports 30, 102277. https://doi.org/10.1016/ j.jasrep.2020.102277.

7. Bochenski ZM (2005) Owls, diurnal raptors and humans: signatures on avian bones. In: O'Connor T (Ed.), Biosphere to Lithosphere: New Studies in Vertebrate Taphonomy [Proceedings of the 9th Conference of the International Council of Archaeozoology, Durham 23-28 August 2002. Oxbow Books, Oxford, pp. 31-45.

8. Bochenski ZM, Tomek T, Tornberg R, Wertz K (2009) Distinguishing nonhuman predation on birds: pattern of damage done by the white-tailed eagle *Haliaetus albicilla*, with comments on the punctures made by the golden eagle *Aquila chrysaetos*. Journal of Archaeological Science 36, 122-129.

9. Bochenski ZM, Tomek T, Wertz K, Kaczanowska M, Kozłowski JK, Sampson A (2018) Who ate the birds: the taphonomy of Sarakenos Cave, Greece. Archaeological and Anthropological Sciences 10 (7), 1603-1615.

10. Bochenski ZM, Wertz K, Tornberg R, Korpimaki VM (2021) How to distinguish duck and wader remains eaten by the peregrine falcon *Falco peregrinus* from those eaten by other birds of prey and humans: a taphonomic analysis. International Journal of Osteoarchaeology 2, 317-326 https://doi.org/10.1002/oa.3067, 10.

11. Bochenski ZM, Tomek T (1997) Preservation of bird bones: erosion versus digestion by owls. International Journal of Osteoarchaeology 7, 372-387.

12. Cáceres I, Bravo P, Esteban M, Expósito I, Saladié P (2002) Fresh and heated bones breakage. An experimental approach. In: De Renzi M, Pardo M, Belinchon M, Peñalver E, Montoya P, Márquez-Aliaga A (Eds.), Current Topics on Taphonomy and Fossilization, pp. 471-479.

13. Chiquet P (2005) Des Mesolithiques amateurs de grenouilles? Une etonnante decouverte sur le site de la Baume d'Ogens (Vaud, Suisse). Paléobiologie 10, 59-67.

14. de Ovalle A (1974) [1646] Histórica relación del reino de Chile. Editorial Universitaria. Santiago.

15. Ericson PG (1987) Interpretations of archaeological bird remains: a taphonomic approach. Journal of Archaeological Science 14(1), 65-75. https://doi.org/10.1016/S0305-4403(87)80006-7.

16. Figueroa R, Corales E (2004) Summer diet comparison between the American Kestrel (*Falco sparverius*) and Aplomado Falcon (*Falco femoralis*) in an agricultural area of Araucanía, Central Chile. Hornero 19(2), 53-60

17. Figueroa R, Alvarado S, Corales E, Shehadeh I (2004) Prey of breeding chilean hawk (*Accipiter chilensis*) in an anden *Nothofagus* forest in northern Patagonia. Wilson Bulletin 116(4), 347-351.

18. Gomez GN (2005) Analysis of bone modifications of *Bubo virginianus*' pellets from Argentina. Journal of Taphonomy 3, 1-16.

19. Gomez GN (2007) Predators' categorization based on taphonomic analysis of micromammals bones: a comparison to proposed models. In: Gutierrez MA, Miotti L, Barrientos G, Mengoni Gonalons GL, Salemme M (Eds.), Taphonomy and Zooarchaeology in Argentina. British Archaeological Reports British Series 1601. Archaeopress, Oxford, 89-103

20. Gomez GN, Kaufmann CA (2007) Taphonomic analysis of *Pseudalopex griseus* (Gray, 1837). Scat assemblages and their archaeological implications. Journal of Taphonomy 5, 59-70.

21. Herrera C, Jaksic F (1980) Feeding ecology of the Barn owl in central Chile and southern Spain: a comparative study. The Auk 97(4), 760-767.

22. Jakisc F, Yáñez J, Schlatter R (1980a) Prey of the Harris' hawk in Central Chile. The Auk 96(1), 196-198.

23. Jakisc F, Schlatter R, Yáñez J (1980b) Feeding ecology of central chilean foxes, *Disicyon culpaeus* and *Dusicyon griseus*. Journal of Mammalogy 61(2), 254-260.

24. Jiménez J, Jaksic F (1989) Behavioral ecology of Grey Eagle-Buzzards, *Geranoaetus melanoleucus*, in Central Chile. The Condor 91, 913-921.

25. Kyselý R (2008) Frogs as a part of the eneolithic diet. Archaeozoological records from the Czech Republic (Kutná Hora-Denemark site, Řivnáč Culture). Journal of Archaeological Science 35, 143-157. https://doi.org/10.1016/j.jas.2007.02.016.

26. Laroulandie V (2002) Damage to pigeon long bones in pellets of the Eagle Owl *Bubo bubo* and food remains of peregrine falcon *Falco peregrinus*: zooarchaeological implications. Acta Zoologica Cracoviensia 45, 331-339.

27. Lizama-Catalán Á, Labarca R (2023) Who eats What: Unravelling a complex taphonomic scenario in the lacustrine deposits of the late Pleistocene archaeological site, Taguatagua 1, central Chile. Quaternary Science Reviews 300, 107831. doi: 10.1016/j.quascirev.2022.107831.

28. Lloveras L, Cosso A, Sole J, Claramunt-López B, Nadal J (2017) Taphonomic signature of golden eagles (*Aquila chrysaetos*) on bone prey remains. Historical Biolology 30:6, 835-854 https://doi.org/10.1080/08912963.2017.1319830.

29. López JM (2020) Actualistic taphonomy of barn owl pellet- derived small mammal bone accumulations in arid environments of South America. Journal of Quaternary Science 35 (8), 1057-1069.

30. López JM, Fernández FJ, Montalvo CI, Chiavazza H, De Santis LJ (2017) The role of the Accipitriformes *Geranoaetus melanoleucus* and *Geranoaetus polyosoma* as small mammal

bones accumulators in modern and archaeological sites from Central Western Argentina. Journal of Taphonomy 15 (1-3), 91-108.

31. Medina-Vogel G, González-Lagos C (2008) Habitat use and diet of endangered southern river otter *Lontra provocax* in a predominantly palustrine wetland in Chile. Wildlife Biology 14(2): 211-220.

32. Mignino J (2021) Tafonomía actualística de huesos de anfibios predados por estrigiformes del centro de Argentina. Contribuciones para estudios paleontológicos y arqueológicos. Spanish Journal of Paleontolology 36 (1). https://doi.org/10.7203/ sjp.36.1.20517.

33. Montalvo CI, Tejerina P (2009) Análisis tafonómico de los huesos de anfibios y roedores depredados por *Athene cunicularia* (Strigiformes, Strigidae) en La Pampa, Argentina. Mamül Mapu: pasado y presente desde la arqueología pampeana 1, 323-334.

34. Montalvo CI, Bisceglia S, Kin MS, Sosa RA (2012) Taphonomic analysis of rodent bone accumulations produced by Geoffroy's cat (*Leopardus geoffroyi*, Carnivora, Felidae) in Central Argentina. Journal of Archaeological Science 39, 1933-1941. https://doi.org/ 10.1016/j.jas.2012.02.024.

35. Montalvo CI, Vezzosi RI, Kin MS (2015) Taphonomic analysis of rodent bones from *Lontra longicaudis* (Mustelidae, Carnivora) scats in fluvial environments. Mastozoología Neotropical 22, 319-333.

36. Montalvo CI, Fernández FJ, Tallade PO (2016) The role of *Bubo virginianus magellanicus* as rodent bone accumulator in archaeological sites: a case study for the Atuel River (Mendoza, Argentina). International Journal of Osteoarchaeology 26 (6), 974-986.

37. Muñoz-Pedreros A Gil C, Yáñez J, Rau J, Möller P (2017) Trophic ecology of two raptors, Barn owl (*Tyto alba*) and Whilte-Tailed kite (*Elanus leucurus*), and possible implications for biological control of hantavirus reservoir in Chile. The Wilson Journal of Ornithology 128(2), 391-403.

38. Pinto-Llona AC, Andrews P (1999) Amphibian taphonomy and its application to the fossil record of Dolina (middle Pleistocene, Atapuerca, Spain). Palaeogeography, Palaeoclimatolology, Palaeoecology 149, 411-429. https://doi.org/ 10.1016/S0031-0182(98)00215-6

39. Rodríguez-Hidalgo A, Saladie P, Marín J, Canals A (2016) Bird-bone modifications by Iberian lynx: a taphonomic analysis of non-ingested red-legged partridge remains. Quaternary International 421, 228-238.

40. Shipman P, Foster G, Schoeninger M (1984) Burnt bones and teeth: an experimental study of colour, morphology, crystal structure and shrinkage. Journal of Archaeological Science 11 (4), 307-325.

41. Simonetti AJ, Cornejo L (1991) Archaeological evidence of rodent consumption in Central Chile. Latin American Antiquity 2 (1), 92-96.

42. Stiner MC, Kuhn SL, Weiner S, Bar-Yosef O (1995) Differential burning, recrystallization, and fragmentation of archaeological bone. Journal of Archaeological Science 22, 223-237

43. Schlatter R, Toro B, Yáñez J, Jaksic F (1980) Prey of the White-Tailed Kite in Central Chile and its relation to the hunting habitat. The Auk 97(1), 186-190.

44. Vélez CM (2019) *Calyptocephalella gayi* (Dumeril y Bibron, 1841). In: Charrier A. (Ed.), Anfibios de los bosques de la zona centro sur y Patagonia de Chile. Corporacion chilena de la Madera, pp. 52 -55.

45. Vélez CM, Acuña PL (2012) Avances en el manejo ex situ de *Calyptocephalella gayi* (rana grande chilena). In: Soto-Azat C, Valenzuela-Sánchez A (Eds.), Conservación de anfibios de Chile, 77-82. Universidad Andrés Bello, Santiago, Chile

46. Weihmüller MP, Brizuela C, Mignino J, Robledo AI. (2022) Bones, carnivores, and grassland fires. Actualistic taphonomy of faunal assemblages from two caves in Central Argentina and its implication for the fossil record. Historical Biology 34,12, 2273-2286. DOI: 10.1080/08912963.2021.2012768
