# Supplementary material for: Taguatagua 3: A new late Pleistocene settlement in a highly suitable lacustrine habitat in central Chile (34°S)
Source: PLoS One. 2024 May 22;19(5):e0302465. doi: 10.1371/journal.pone.0302465 (PMC11111044; doi:10.1371/journal.pone.0302465)
Supplement: S4 File — (PDF) [file pone.0302465.s022.pdf]

Supplementary File 4
Stable isotopes analysis from gomphothere tooth (bioapatite) from TT-3 site

Methodology

Five samples of bioapatite were analyzed from the dental enamel of the gomphothere recovered from TT-3. Tooth surfaces were first cleaned with a tungsten abrasive drill bit. The samples of enamel were removed by drilling with a diamond bit. A rotary hand drill with a diamond-tipped dental burr was used to recover enamel from an area of the tooth as large as possible to avoid seasonal bias at the time of mineralization. Powdered enamel samples were chemically treated at the Biomarkers Laboratory of the Institut Català de Paleoecologia Humana i Evolució Social (IPHES-CERCA) (González-Guarda et al., 2022). Samples weighed from 3.5 mg to 9.5 mg. Chemical treatment of samples was based on protocols originally proposed by Koch et al., (1997) that were modified in Tornero et al., (2013). Samples were treated for 4 h in 0.1 M acetic acid [CH_3COOH] (0.1 ml solution/0.1 mg of sample), neutralized with distilled water, and freeze-dried. Pretreated powders were analyzed individually on a Thermo Kiel III device interfaced with a MAT Finnagan 253 at the Scientific and Technological centers of the University of Barcelona (CCiTUB), Spain. The samples were reacted in a vacuum with 100% phosphoric acid [H_3PO_4] at 70°C in individual vessels and purified in an automated cryogenic distillation system. $\delta^{13}C$ values are expressed relative to VPDB. Accuracy and precision of the measurements were checked using two internal laboratory calcium carbonate standards (RC-1 and CECC) normalized to NBS18 and NBS19 international standards. A total of 16 RC-1 and CECC samples were measured (RC-1 expected values +2.83‰ for $\delta^{13}C$; CECC expected values −20.78‰ for $\delta^{13}C$). The mean analytical precision of RC-1 was +0.01‰ for $\delta^{13}C$ values and +0.02‰ for $\delta^{18}O$. Stable isotope results are reported in the δ-notation $\delta^{H}X_{sample}=[(R_{sample}-R_{standard})/R_{standard}]x1000$, where X is the element, H is the mass of the rare, heavy isotope, and $R={}^{13}C/{}^{12}C$, or ${}^{18}O/{}^{16}O$. $\delta^{13}C$ and $\delta^{18}O$ values are expressed in the Vienna-Pee Dee Belemnite (VPDB) standard, although $\delta^{18}O$ values are also given in terms of the VSMOW (Vienna Standard Mean Ocean Water) standard, so that VPDB values can be converted into VSMOW applying the following formula: $\delta^{18}O_{SMOW} = (1.0309 \times \delta^{18}O_{VPDB}) + 30.909$.

In this study, a $\delta^{13}C_{atmCO2}$ value of −6.5‰ was used because it is an accepted value for late Pleistocene studies (Tipple et al., 2010). Therefore, stable isotope data of modern vegetation were corrected because the modern composition of ${}^{13}C_{atmCO2}$ has a value of −8‰ (Marino and McElroy, 1991). Since it has been suggested that body mass (bm) has an effect on the physiological values of carbon enrichment (see Tejada-Lara et al., 2018), the equation $\varepsilon^{*} = 2.4 + 0.034$ (bm) was applied to obtain the enrichment between bioapatite and the diet of *Notiomastodon platensis* ($\varepsilon^{*}_{diet\text{-}bioapatite}$). When obtaining the $\varepsilon^{*}_{diet\text{-}bioapatite}$ ($\delta^{13}C$ = 15‰) value of *N. platensis* (bm: 6.000 kg), it was possible to increase the confidence of the results obtained from the comparisons between $\delta^{13}C_{bioapatite}$ values of mammals with different body mass (bm) (Tejada-Lara et al., 2018).

We first estimated the $\delta^{18}O_{meteoric\ water\ (mw)}$ value ingested by gomphotheres using their enamel $\delta^{18}O_{PO4}$ values and then applied the $\delta^{18}O_{mw}$-$\delta^{18}O_{PO4}$ linear regression established for their nearest-living relatives: modern elephants. Such equation was selected assuming that there are no significant differences in the fractionation factor between $\delta^{18}O_{PO4}$ and $\delta^{18}O_{mw}$ of extinct gomphotheres and extant elephants. The equation used was the following: $\delta^{18}O_{mw\ (VSMOW)}$ = ($\delta^{18}O_{PO4\ (VSMOW)}$ - 23.3)/0.94 (Ayliffe et al., 1992).

Results

When obtaining the $\mathcal{E}^{*}_{diet\text{-}bioapatite}$ ($\delta^{13}C$ = 15‰), we get a mean value of -28‰ (Table 1), which is an estimated minimum value for individuals with an important compound of closed canopy plants in diet (e.g., González-Guarda et al., 2018; Hofman-Kamińska et al., 2018). Although grass consumption should not be completely ruled out, since the mean value of the values (δ 13 C = -13 ± 0.7‰; table 2) falls within the isotopic ranges proposed by Domingo et al., (2012) for an environment that also includes non-arboreal elements (i.e. −14.5‰ to −9.5‰; wooded C 3 grassland to open). The δ 18 O mw values (δ 18 O mw = -6.8 ± 1.3‰) (Table 3) from sampled gomphotheres indicates paleoenvironments with similar proportion of meteoric water than current times (current range for the north central area of Chile: ca. -3‰ to -10‰; according to the Global Network of Isotopes in Precipitation: GNIP).
Consequently, the gomphothere from TT-3 was consuming mainly arboreal vegetation, under a paleoclimate and paleoenvironment scenario very close to the current one (i.e. Mediterranean climate); that is, relatively warmer and more arid conditions than those described above for the end of the Pleistocene (e.g., Valero-Garcés et al., 2005).

**Laboratory code**	**Samples**	**$\delta^{13}C$ (‰ VPDB)**	**$\mathcal{E}^{*}_{diet\text{-}bioapatite}$ (+15‰)**
**TT3 /1/K1485**	Unit C5-C7-N18	-12.59	-27.59
**TT3/2/K1485**	Unit C4-C7-N19-20	-14.35	-29.35
**TT3/3/K1485**	Unit C4-C7-N20-1	-12.89	-27.89
**TT3/4/K1485**	Unit C5-C7-N18	-12.42	-27.42
**TT3/5/K1485**	Unit C4-C7-N19-20	-12.79	-27.79

Table 1. $\delta^{13}C$ values in the bioapatite of dental enamel of Gomphotheriidae undet. from TT-3.

**$\delta^{13}C$ (‰ PDB)**					
**Taxon**	**N**	**Max**	**Min**	**Mean**	(σ)

**Gomphotheriidae indet.**	5	-12.4	-14.4	-13	0.7
**$\delta^{18}O$ (‰VSMOW)**					
**Gomphotheriidae indet.**	5	-3.3	-6.8	-4.9	1.3

Table 2. $\delta^{13}C$ enamel isotopic results from Gomphotheriidae undet. from TT-3. Number of samples: n; maximum: Max; minimum: Min; mean values: Mean; standard deviation: σ.

Laboratory code	Samples	$\delta^{18}O_{CO3}$ (‰ VPDB)	$\delta^{18}O_{CO3}$ (‰ VSMOW)	$\delta^{18}O_{PO4}$ (‰ VSMOW)	$\delta^{18}O_{mw}$ (‰ VSMOW)
TT3 /1/K1485	Unit C5-C7-N18	-3.3	27.5	18.4	-5.21
TT3/2/K1485	Unit C4-C7-N19-20	-5.1	25.6	16.5	-7.23
TT3/3/K1485	Unit C4-C7-N20-1	-6.8	23.9	14.9	-8.93
TT3/4/K1485	Unit C5-C7-N18	-4.7	26.1	17	-6.6
TT3/5/K1485	Unit C4-C7-N19-20	-4.5	26.3	17.2	-6.4

Table 3. $\delta^{18}O$ values in the bioapatite of dental enamel of of Gomphotheriidae undet. from TT-3 Bioapatite $\delta^{18}O$ of carbonate fraction ($\delta^{18}O_{CO3}$); bioapatite $\delta^{18}O$ of phosphate fraction ($\delta^{18}O_{PO4}$); meteoric water ($\delta^{18}O_{mw}$).

References

1. Ayliffe LK, Lister AM, Chivas AR. The preservation of glacial interglacial climatic signatures in the oxygen isotopes of elephant skeletal phosphate. Palaeogeography, Palaeoclimatology, Palaeoecology. 1992; 99, 179–191.

2. Domingo L, Prado JL, Alberdi MT. The effect of paleoecology and paleobiogeography on stable isotopes of Quaternary mammals from South America. Quaternary Science Reviews. 2012; 55, 103-113.

3. Hofman-Kamińska E, Bocherens H, Borowik T, Drucker DG, Kowalczyk R. Stable isotope signatures of large herbivore foraging habitats across Europe. PLoS One. (2018; 13(1), e0190723.

4. Koch PL, Tuross N, Fogel ML. The effects of sample treatment and diagenesis on the isotopic integrity of carbonate in biogenic hydroxylapatite. Journal of Archaeological Science. 1997; 24, 417–429.

5. Marino BD, McElroy MB. Isotopic composition of atmospheric CO2 inferred from carbon in C_4 plant cellulose. Nature. 1991; 349(6305), 127-131.

6. Tejada-Lara JV, MacFadden BJ, Bermúdez L, Rojas G, Salas-Gismondi R, Flynn JJ. Body mass predicts isotope enrichment in herbivorous mammals. Proceedings of the Royal Society B. 2018; 285(1881), 20181020.

7. Tipple BJ, Meyers SR, Pagani M. Carbon isotope ratio of Cenozoic CO2: A comparative evaluation of available geochemical proxies. Paleoceanography. 2010; 25(3). doi:10.1029/2009PA001851

8. Tornero C, Bălăşescu A, Ughetto-Monfrin J, Voinea V, Balasse M Seasonality and season of birth in early Eneolithic sheep from Cheia (Romania): methodological advances and implications for animal economy. Journal of Archaeological Science. 2013; 40(11), 4039-4055.
